# DURABLE: A Workflow for Determining Corrosion-Driving and Protective Microbial Mechanisms

**DOI:** 10.1021/acs.est.6c07071

**Published:** 2026-08-11

**Authors:** Aifen Zhou, Yuwen Li, Han Zhang, Evelyn Callaway, Song-I Han, Jason S. Lee, Kyongbum Lee, Arul Jayaraman, Paul de Figueiredo, Arum Han

**Affiliations:** † Department of Chemical Engineering, 2655 Texas A&M University, College Station, Texas 77843, United States; ‡ Department of Electrical and Computer Engineering, Texas A&M University, College Station, Texas 77843, United States; § U.S. Naval Research Laboratory, Stennis Space Center, Stennis Space Center, Mississippi MS39529, United States; ∥ Department of Chemical and Biological Engineering, 1810 Tufts University, Medford, Massachusetts 02155, United States; ⊥ Department of Biomedical Engineering, Texas A&M University, College Station, Texas 77843, United States; # Christopher S Bond Life Sciences Center, 14716 University of Missouri, Columbia, Missouri 65211, United States; ∇ Department of Molecular Microbiology and Immunology, University of Missouri School of Medicine, Columbia, Missouri 65211, United States; ○ Department of Veterinary Pathobiology, University of Missouri School of Veterinary Medicine, Columbia, Missouri 65211, United States

**Keywords:** microbiologically influenced corrosion (MIC), microbial community composition, fuel storage tank, rapid carbon steel bead-based corrosion phenotype screening, bacterial strain isolation, corrosion metabolites profiling

## Abstract

Microbiologically influenced corrosion (MIC) threatens global infrastructure, causing billions of dollars in annual losses. Its persistence stems from unresolved mechanisms—particularly the metabolites produced by microorganisms that drive or inhibit corrosion—and the microbial community structures. Progress has been hindered by the absence of systematic workflows to rapidly and accurately identify MIC-relevant microorganisms and their functions. Here, we present **DURABLE** (**D**etection of **U**nique Corrosion **R**esistant or **A**ccelerating **B**iologics in a **L**aboratory **E**nvironment), a pipeline that couples high-throughput microbial screening with genomic and metabolic workflows. We applied the DURABLE workflow to six diesel tank samples and revealed fuel-dependent microbial community structures, which showed greater diversity and evenness in bacterial communities than their fungal counterparts. The workflow used carbon steel beads to rapidly screen over 80 bacterial isolates for corrosive activity, reducing assay time to approximately 2 days compared with the conventional 30-day metal coupon test. More than 40 isolates were identified as corrosive. Further testing using mass spectrometry analysis revealed corrosion-associated metabolites, which were further validated using electrochemical assays. Thus, DURABLE achieved a ∼15-fold increase in screening speed and provided a scalable and mechanistic framework for dissecting MIC dynamics. We expect this advance will enable the development of precision mitigation strategies in hydrocarbon fuel infrastructure.

## Graphical abstract

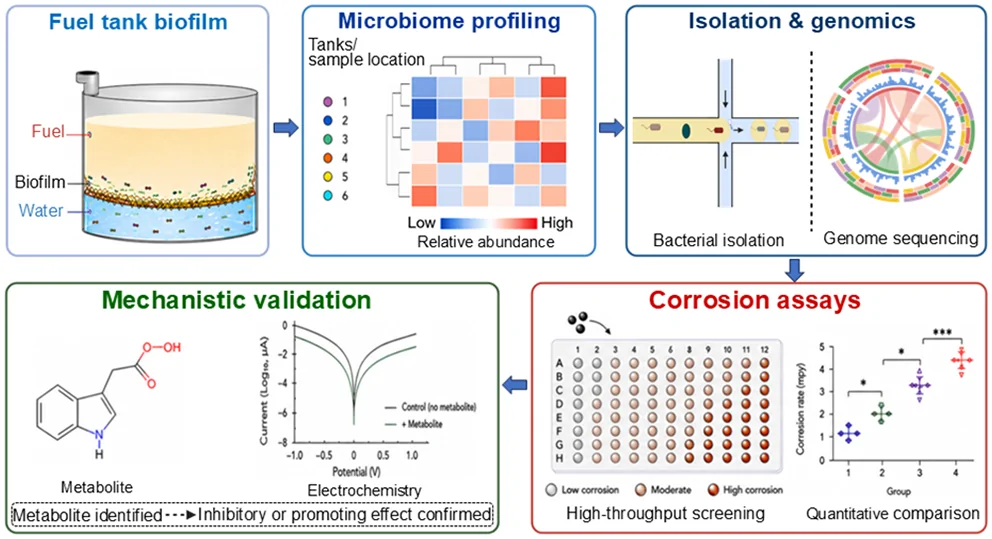

## Introduction

Microbiologically influenced corrosion (MIC) is a significant and costly problem across a wide range of industries, including oil and gas, wastewater, infrastructure, marine, food, and defense^1,2^ . For example, in the oil and gas industry, corrosion of fuel tanks is a serious and growing concern, leading to several billion dollars in annual losses.^1^ Conventional MIC assessments include visual inspection, analysis of corrosion products, and corrosion rate measurements using metal coupons, while advanced technologies such as DNA-based analysis coupled with electrochemical technologies enable the identification of microorganisms associated with MIC.^3–6^

Despite the success of these approaches, several major gaps remain. First, there is a lack of integrated high-throughput systems for phenotypical screening and isolation of MIC-associated microorganisms. Therefore, existing methods are labor-intensive, time-consuming and offer limited mechanistic insight. Second, there is an absence of an integrated analytical framework linking the microbial community composition, individual microbial functions, and metabolic profiles to corrosion. Although MIC is known to result from chemical and electrochemical processes driven by microbial metabolites, these elements have rarely been studied in a comprehensive and unified manner. Most studies focus on characterizing microbial community compositions, derived enrichment cultures, or isolated bacterial strains,^7–10^ often using amplicon sequencing with limited sequencing depth and minimal characterization of individual microorganisms. To address these limitations, we developed DURABLE (**D**etection of **U**nique corrosion **R**esistant or **A**ccelerating **B**iologics in a **L**aboratory **E**nvironment), an integrated pipeline that couples high-throughput microbial screening with genomic and metabolomic workflows to accelerate MIC mechanism characterization.

The DURABLE pipeline follows a systematic approach that includes microbial community analysis, bacterial isolation, and molecular characterization to identify key microorganisms associated with MIC and the metabolites they produce, all at high throughput. We applied the DURABLE workflow to analyze MIC in fuel tank systems, which serve as an ideal testbed where the intrinsic water and fuel phases support microbial growth. Moreover, the MIC risk in fuel tanks is often intensified by the increased use of renewable fuel blends, which are more susceptible to microbial activities. Many aspects of MIC in fuel-storage tanks remain unsolved. First, the heterogeneity of microbial community composition in the diesel tank fuel phase and water phase has been reported.^9^ Therefore, it is important to explore the spatial variance of microbial communities within fuel tanks and how different fuel types influence these communities. Second, the role of fuel tank microorganisms and whether they promote or prevent MIC is still largely unknown. At the individual strain level, research has mainly focused on a few model MIC strains such as sulfate reducers, acetogens, and iron oxidizers, typically studied under well-defined laboratory conditions.^11–13^ A significant disconnect remains between laboratory findings and real-world observations due to the limited availability of information on the relationship between fuel tank MIC-relevant microbial isolates and their corrosivity in real-world environments. Meanwhile, studies of hydrocarbon-degrading bacterial strains or communities have primarily focused on bioremediation and fuel biodeterioration,^14,15^ leaving their potential role in MIC largely unexplored, except for the isolation of two fungal strains from B20 storage tanks that have been shown capable of degrading biodiesel and linked to higher corrosion rates as well as pitting corrosion under controlled conditions.^16^ Therefore, isolating and characterizing fuel tank MIC-relevant microorganisms constitutes an essential first step for further advancing the field.

We applied the DURABLE workflow to fuel tank biofilm samples to isolate many previously unknown corrosion-associated microorganisms, tested their individual corrosion characteristics, and then identified and validated key MIC-driving or protective metabolites. Here, a 96-well carbon steel bead-based high-throughput corrosion assay was used to significantly improve the corrosion phenotype screening efficiency as well as individual strain corrosivity characterization. This work was followed by mass spectrometry analyses to map corrosion-associated metabolites to microbial strains of interest, followed by validation of the potential key metabolites through electrochemical analyses. Taken together, this work establishes DURABLE as an integrated pipeline for characterizing MICs.

## Results

### The DURABLE Workflow

To obtain a comprehensive understanding of the microbial communities in biofilm samples from real-world fuel storage tanks, we isolated and characterized bacteria associated with microbial-influenced corrosion (MIC) using the DURABLE workflow (Figure 1). **First**, bacterial strains were isolated from samples harvested from two distinct fuel tank locations with microbial contaminations (Figure S1) using a newly developed agarose gel droplet microfluidics-based microbial cocultivation platform. In this system, individual microbial cells are encapsulated within porous agarose microspheres at single-cell resolution, allowing cell-to-cell communication and better preservation of microbial diversity compared to conventional plating methods.^17^ This gel-droplet-based cultivation approach allows single-strain-resolution isolation of microorganisms, thereby enhancing opportunities to discover novel MIC-associated bacteria. Alongside bacterial isolation, culture-independent 16S rRNA and ITS amplicon sequencing was conducted to analyze bacterial and fungal community structures of samples harvested from various locations within the fuel tanks. Alpha and beta diversities were also analyzed to interpret the relationships among the fuel type, sampling location, and microbial community composition. **Second**, corrosion phenotypes of bacterial isolates from the first step were assessed by using a 96-well screening platform with carbon steel (CS) beads as corrosion substrates. Rapid medium discoloration in each well served as a proxy for metal corrosion, significantly reducing the time required to assess this feature. **Third**, the corrosive activities of the selected corrosive and noncorrosive bacterial strains were evaluated using conventional weight loss experiments with CS coupons under water or water/fuel/air conditions that mimic real-world scenarios. These tests validated outcomes from the 96-well plate corrosion phenotype screening approach and quantified the corrosion rates of the isolated strains under realistic conditions. **Fourth**, we analyzed the whole-genome sequences and metabolite profiles of the selected MIC-relevant bacterial strains to determine their taxonomy, genetic potential, and ability to utilize fuel as a carbon source. **Finally**, key metabolites promoting or inhibiting MIC were identified via comparative metabolomics and validated by electrochemical analysis using standard compounds.

**Figure 1:**
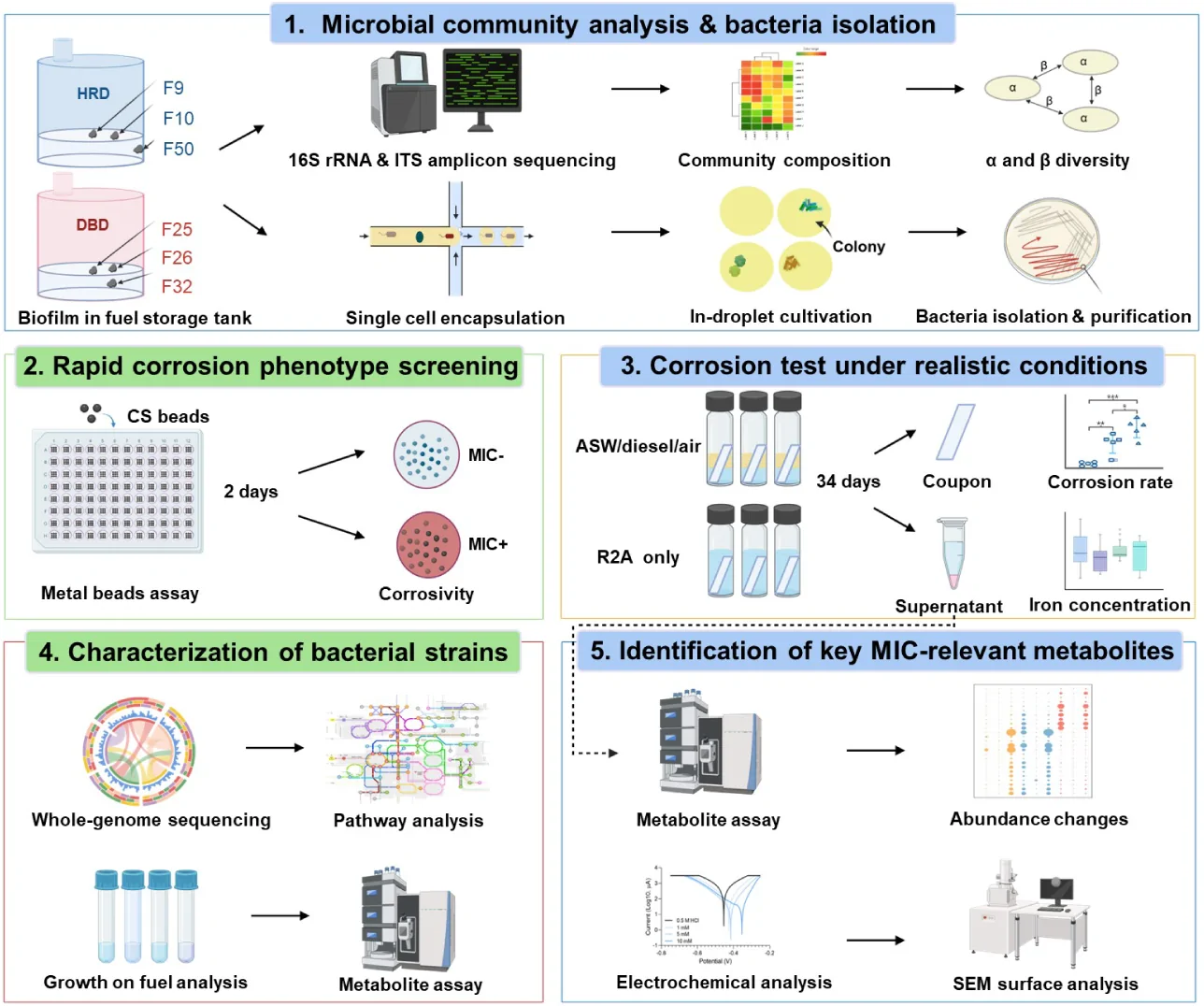
DURABLE workflow. Microbial communities in six fuel tank samples were analyzed using 16S rRNA and ITS amplicon sequencing. In parallel, microbial cells were encapsulated in agarose gel droplets at single-cell resolution, amplified within the gel droplets, and then recovered onto agar plates at single-strain resolution. Corrosion characteristics of each strain were screened using a carbon steel (CS) bead-based 96-well plate assay. Representative bacterial strains were selected for CS coupon corrosion tests under more realistic conditions. Whole genome sequencing was conducted to reveal the strain identity, followed by pathway analysis, growth assays, and metabolite assays. Key metabolites promoting or preventing MIC were identified via comparative metabolite analyses and validated with electrochemical analyses. HRD: hydrogenated renewable diesel; F76: petroleum-based Navy F76 diesel.

### Fuel Type and Location-Dependent Bacterial Communities

To test whether bacterial community composition varies by fuel type and location within storage tanks, we applied the first step of the DURABLE workflow to six samples harvested from distinct locations in two tanks storing either hydro-processed renewable diesel (HRD) or petroleum-based Navy F76 diesel (F76). We found that bacterial community composition was fuel type- and location-dependent. A sufficient number of qualified 16S rRNA amplicon sequencing reads per sample (32.5 to 131.6K) were obtained to assess bacterial diversity (Figure S2a). Species richness was similar between the HRD and F76 tanks (138 ± 29 vs 120 ± 53, *P* = 0.63), but the HRD tank had a significantly higher Shannon index than the F76 tank (5.3 ± 0.3 vs 3.3 ± 0.1; *P* = 0.0004), indicating greater community evenness (Figure S3a,b). Taxonomy compositions were clustered by tank and sampling location, with interface samples clustered together (Figure 2a,b). Beta diversity analysis showed clear separation by tank and lower variance among HRD tank samples (Figure 2c), supported by Bray-Curtis dissimilarity analyses (Figure S3c). *Proteobacteria* was the dominant phylum across all samples, consistent with previous findings in diesel storage tanks,^6^ on diesel and gasoline car tank lids,^10^ and in B20 (diesel blended with 20% biodiesel) diesel tanks.^9^

**Figure 2:**
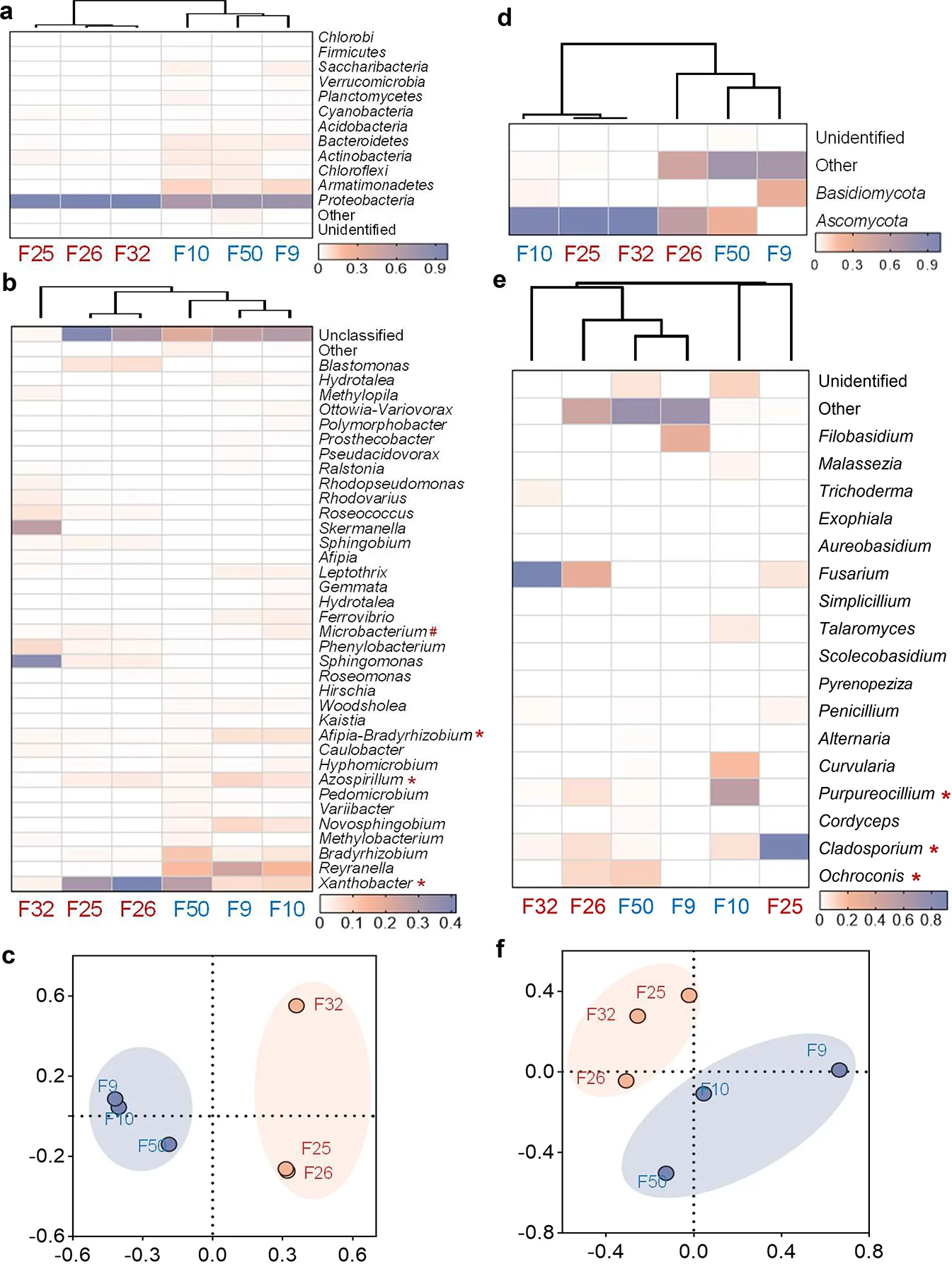
Taxonomy heatmaps with cluster analysis of the community compositions. a. Phylum-level bacterial community compositions. b. Genus-level bacterial community compositions. c. Beta diversities of bacterial communities at the genus level. d. Fungal community compositions at the phylum level. e. Genus-level fungal community compositions. f. Beta diversities of fungal communities at the genus level. #: Multiple strains were isolated from this genus. *: High relative abundances in samples from both tanks. Blue font: Samples from the HRD tank. Red red font: Samples from the F76 diesel tank.

At the genus level, distinct dominant genera (average relative abundance of >4%) were observed in each tank. In the HRD tank, *Reyranella* (17.3%), *Xanthobacter* (14.3%), *Bradyrhizobium* (6.3%), *Azospirillum* (5.0%), and *Novosphingobium* (4.9%) were dominant. In the F76 tank, *Xanthobacter* (25.2%), *Sphingomonas* (14.71%), and *Skermanella* (9.1%) were the most dominant. *Xanthobacter* was the dominant genus shared between tanks, while *Novosphingobium* and *Skermanella* were unique to the HRD and F76 tanks, respectively. Other shared genera (average relative abundance >1%) included *Azospirillum* and *Microbacterium.* Among these, *Xanthobacter sp.* had been isolated from crude oil tank sludge,^18^ *Azospirillum* was found dominant in bioremediation microcosms with creosote-contaminated soil,^19^ while *Microbacterium* was not detected in 16S rRNA amplicon-based fuel storage tank microbiomes in the literature. Different dominant genera have been found in tanks storing other fuel types, for example, *Acetobacter* and *Denitratisoma* in ethanol tanks,^8^ *Pannonibacter* and *Nitrospirillum* in B20 biodiesel tanks in the southeast USA,^9^ *Acetobacteraceae* and *Clostridiaceae* group 1 in B20 biodiesel tanks in the southwest USA,^9^ and *Prauseria* and *Bacillus* in crude oil storage tanks,^20^ highlighting the strong influence of fuel type on microbial communities.

### Fungal Community Composition

Fungal diversity was reported to be lower than bacterial diversity in B20 tanks.^9^ To test whether this pattern also holds for other fuel types, we analyzed fungal communities in six samples during the first step of the DURABLE workflow. A sufficient number of qualified ITS amplicon sequencing reads per sample (12 to 205K) were obtained to assess fungal diversity (Figure S2b). No significant differences were observed in the number of fungal species (32 ± 22 in the HRD tank vs 16 ± 4 in the F76 tank, *P* = 0.34) or the Shannon index (2.6 ± 0.4 for the HRD tank vs 1.9 ± 1.1 for the F76 tank; *P* = 0.36) (Figure S3d,e). Samples from the same tank were not clustered taxonomically (Figure 2d,e), nor were they separated by tank in beta diversity plots (Figure 2f), consistent with their high Bray-Curtis dissimilarities (Figure S3f). *Ascomycota* was the most dominant phylum across all samples. Dominant genera (average relative abundance >4%) included *Purpureocillium* (18.0%), *Curvularia* (6.3%), and *Ochroconis* (4.2%) in the HRD tank, *and Fusarium* (42.5%) and *Cladosporium* (33.8%) in the F76 tank. *Curvularia* was unique to the HRD tank, while *Fusarium* was nearly exclusive to the F76 tank (0.02% relative abundance in the HRD tank). Lower levels of *Cladosporium* in the HRD tank (3.2%), *Purpureocillium* (2.82%), and *Ochroconis* (3.45%) were also detected in the F76 tank. *Cladosporium sp.* has been isolated from kerosene storage tanks^21^ and detected in diesel and biodiesel storage tanks,^7^ while *Purpureocillium* and *Ochroconis* have not, although *Purpureocillium sp.* has been previously isolated from engine oil-contaminated soil.^22^ Consistent with previous reports in B20 tanks,^9^ fungal diversity was lower than bacterial diversity in both the F76 tank and the HRD tank, suggesting that fungi and bacteria play distinct roles in fuel storage tank conditions..

### Rapid Metal Corrosion Phenotype Screening Using CS Beads

Our community analysis revealed substantial phylogenetic diversity, which is often linked to functional diversity.^23^ To test the hypothesis that the fuel tank bacterial community contained both corrosive and noncorrosive members, we implemented the first two steps of the DURABLE workflow to isolate bacterial strains and then rapidly screened their metal corrosion phenotypes using a CS bead-based colorimetric assay. Following the evaluation of microbial cell viability (Figure S4), an agarose gel droplet encapsulation-based single-strain isolation method was employed to isolate bacterial strains (Figure S5). A total of 40 isolates were obtained from sample F50 (HRD tank) and 44 from sample F32 (F76 tank). To assess their corrosion phenotypes, we developed a 96-well plate colorimetric assay utilizing CS beads (diameter: 500 μm). We hypothesized that the smaller size and higher surface area of CS beads would speed up phenotypic testing of microbial corrosivity compared to conventional CS coupon weight loss-based corrosion rate testing, with changes in the color of the media serving as a proxy for corrosion activity (Figure 3a). By day two, a brownish color change appeared and intensified over time (Figure S6), with microscopy images confirming rust in discolored wells while none was present in clear wells (Figure 3b). No color change or rust formation was observed in the negative control without bacteria. Compared to the conventional month-long metal coupon assay, the CS bead-based 96-well assay was ∼15× faster, and thus enabled high-throughput corrosivity screening of large numbers of microbial isolates. Among the bacterial isolates tested, half (42) were corrosive and the remainder (42) were noncorrosive.

**Figure 3:**
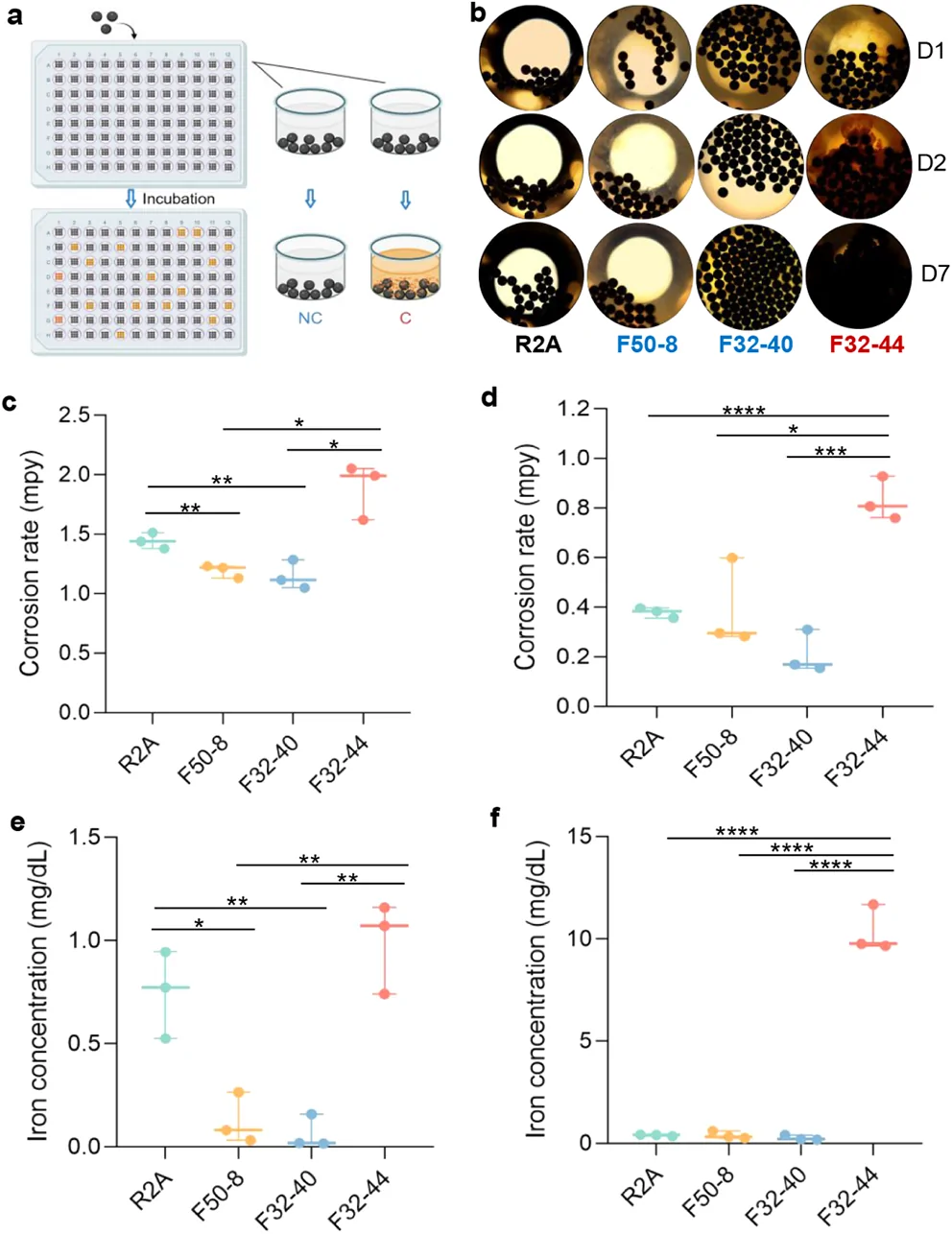
Assessment of the corrosion phenotypes of bacterial isolates. a. Metal corrosion phenotype screening using CS beads in a 96-well plate format. NC, noncorrosive; C, corrosive. b. Microscopy images of the CS beads in the wells. The color of wells with corrosive activity turns brownish on day 2 and dark brown on day 7. c. Average corrosion rates after exposure to conditions mimicking the fuel tank environment (ASW/diesel/air) for 34 days. d. Average corrosion rates after exposure to the aqueous phase only (R2A) for 34 days. e. Iron concentrations in supernatants at the end of exposure to ASW/fuel/air conditions for 34 days. f. Iron concentrations in supernatants at the end of exposure to R2A for 34 days. Unpaired *t*-test: *: *P* < 0.05; **: *P* < 0.01; ***: *P* < 0.001; ****: *P* < 0.0001.

### Metal Corrosion Rate Assessment Using CS Coupons

Based on distinct corrosion phenotypes from the CS bead assay, two noncorrosive strains (F50-8, F32-40) and one potentially corrosive strain (F32-44) were selected for further testing under simulated fuel tank conditions in the third step of the DURABLE workflow. Corrosion rates (CRs) were measured using CS coupons under either an aqueous phase (R2A medium) or ASW/diesel/air conditions to mimic the fuel storage tank environments (Figure S7a). ASW lacks a carbon source and contains minimal mineral salts.^16^ Incubation with R2A alone (without added bacteria) was used as a negative control (NC). Under ASW/fuel/air, CRs of the corrosive strain F32-44 and NC were significantly higher than those of noncorrosive strains, with no difference between F32-44 and NC (Figure 3c). In R2A, F32-44 showed significantly higher CRs than all other groups, with no differences among the noncorrosive strains and NC (Figure 3d). These results not only confirmed the validity of the CS bead-based rapid corrosivity phenotyping results but also demonstrated the influence of HRD and F76 on bacterial metabolism and MIC activity.

Iron concentrations in the supernatants at the end of the experiment showed similar trends to the CR. Specifically, under ASW/diesel/air conditions (Figure 3e), both the corrosive strain F32-44 and NC showed significantly higher iron concentrations than the noncorrosive strains F50-8 and 32-40, while no differences were observed between the two noncorrosive strains, as well as between the corrosive strain F32-44 and NC. In R2A (Figure 3f), the corrosive strain F32-44 showed a significantly higher iron concentration than all other groups. Overall, higher corrosion rates but lower iron concentrations were observed under ASW/diesel/air conditions, possibly due to the formation of insoluble iron­(III) oxide in oxygen-rich environments compared to more soluble iron­(II) in the fully submerged R2A-only condition. Importantly, the data shown here indicate that soluble iron concentration is another reliable proxy for corrosion phenotype assessment.

### Identity and Morphology of the Representative Corrosive and Noncorrosive Strains

Our corrosion tests revealed that these bacterial isolates possess either corrosive or noncorrosive activities. To determine their taxonomy and genetic basis for growth in fuel tanks, we performed whole-genome sequencing and morphological analyses (the fourth step of the DURABLE workflow). The three representative strains exhibited distinct genomic features (Table S1), where F32-40 and F32-44 exhibited higher GC content (>70%) than F50-8 (43%). Notably, F50-8 harbored seven copies of the 16S rRNA gene, compared to two in F32-40 and three in F32-44, suggesting a potentially higher growth rate. Full-length 16S rRNA sequence-based phylogenetic trees were built using reference genomes (Figure 4). F32-44 is closely related to *Cellulosimicrobium cellulans* ORNL-0100. A soil isolate, *C. cellulans* CWS2, is a pleomorphic, Gram-positive, facultative anaerobe capable of degrading benzo­(a)­pyrene (BaP).^24^ F50-8 is related to *Acinetobacter lwoffii* H7, a Gram-negative, aerobic, chemoheterotroph; other *Acinetobacter sp.* isolated from oil sludge enrichment were shown to degrade petroleum hydrocarbons.^25^ F32-40 is closely related to *Microbacterium proteolyticum* CECT 8356, a Gram-positive, aerobic endophyte isolated from saltmarsh plant roots.^26^

**Figure 4:**
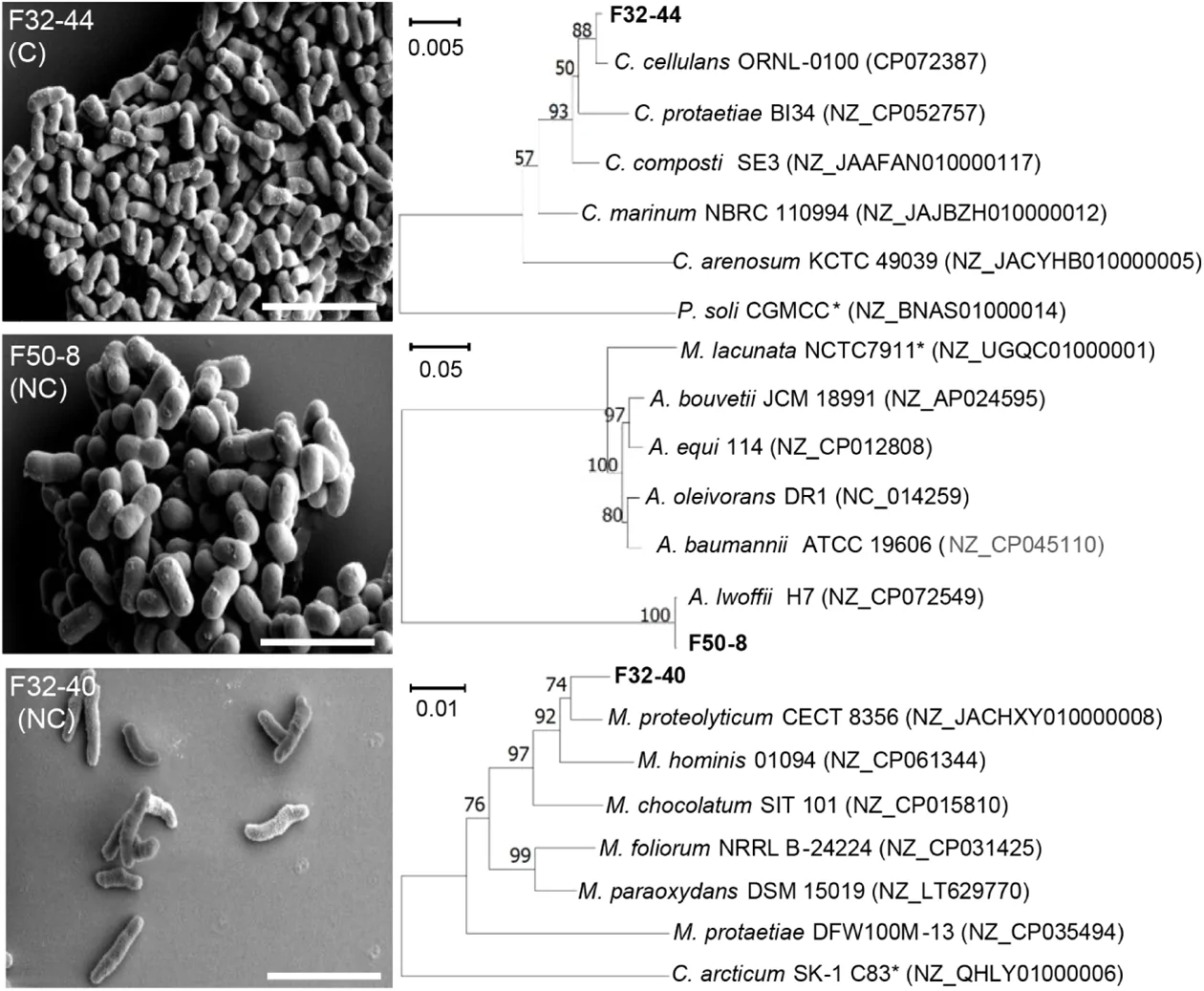
Characterization of the representative corrosive and noncorrosive strains. **Left** panel: Bacterial morphology analyzed using scanning electron micrographs (scale bar: 3 μm). **Right** panel: Phylogenetic trees based on full-length 16S rRNA sequences. Reference genomes from the NCBI genome database were used for alignment and phylogenetic tree construction. *: Outgroup. C, corrosive; NC, noncorrosive.

Colony and cell morphologies were examined on both R2A plates (Figure S8a) and in liquid cultures (Figure 4). F32-44 exhibited rod-shaped cells (∼1 μm × 0.3 μm) and formed yellow colonies, resembling *C. cellulans* from biodiesel-contaminated soils.^27^ F50-8 exhibited short rod cells (∼0.75 μm × 0.5 μm) and formed pale white colonies, similar to *A. lwoffii* isolated from fish.^28^ F32-40 exhibited longer rod cells (1.5 ∼ 2 μm × 0.3 μm) and formed light brown colonies. *Cellulosimicrobiums* and *Microbacterium* strains were previously obtained from car tank lid microbiome enrichments, although undetected in 16S rRNA amplicon sequencing data.^10^ Similarly, *Cellulosimicrobiums* and *Acinetobacter* were not detected in this study’s 16S rRNA amplicon sequencing data; however, multiple *Cellulosimicrobiums* and *Microbacterium* strains were isolated from both fuel tanks, and genomic variations were observed among these strains (Table S1). In addition, *Acinetobacter* was detected in a later batch of fresh samples from the same fuel tanks (Figure S9). These findings highlight the effectiveness of the agarose gel droplet microfluidics-based method for isolating diverse fuel storage tank bacterial strains.

### Fuel Degradation and Biofilm Formation-Related Genes in Genomes

After identifying representative strains, we assessed whether their functional diversity corresponded to variation in genes involved in fuel degradation and/or biofilm formation, which are key traits for survival and promotion of corrosion in fuel tanks. As part of the fourth step of the DURABLE workflow, we analyzed the abundance of these genes. As shown in Figure 5, all strains harbored alcohol dehydrogenase and aldehyde dehydrogenase for aliphatic hydrocarbon degradation,^29,30^ while the alkane 1-monooxygenase gene was found exclusively in *Acinetobacter* F50-8, suggesting that interspecies metabolic cooperation^31^ supports their survival in fuel storage tank conditions. For aromatic hydrocarbon degradation, bacteria typically use two major pathways through central intermediates, catechol or protocatechuate, which are further metabolized via ortho or meta ring cleavage into the TCA cycle.^32^ Ortho-cleavage enzymes of protocatechuate are common in *Actinobacteria* and *Proteobacteria*, while the meta-cleavage enzymes are mostly found in *Proteobacteria*.^33^ Notably, *Microbacterium* F32-40, an *Actinobacteria*, carries genes for all four ring-cleavage pathways; F50-8 has ortho-cleavage genes for both intermediates, while F32-44 has only catechol meta-cleavage genes, highlighting functional diversity and potential complementary roles in aromatic hydrocarbon degradation among these strains.

**Figure 5:**
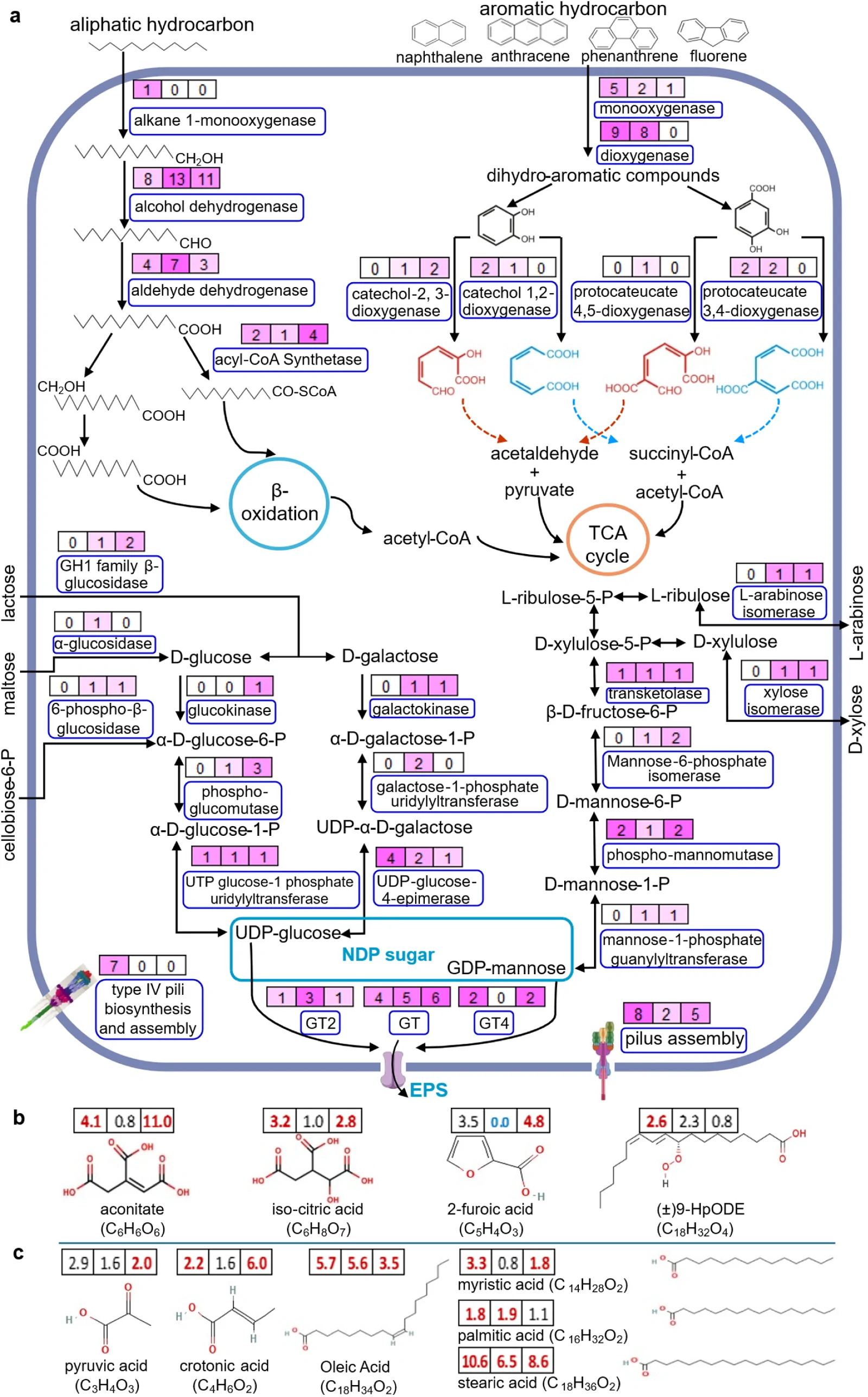
Potential metabolic pathways in the representative strain genomes and the metabolites in the cell culture supernatants with significant abundance changes when using 1% fuel as a carbon source: a. Genes involved in the degradation of aliphatic or aromatic hydrocarbons, biosynthesis of nucleotide sugars (NDP sugars) for EPS production, pilus assembly, and type IV pilus biosynthesis and assembly in the genomes. Color-coded numbers indicate the number of genes in F50-8, F32-40, and F32-44 genomes, from left to right. GT, glycosyltransferase; GT2, glycosyltransferase family 2; GT4, glycosyltransferase family 4. b. Metabolites in supernatants of cell cultures using 1% diesel blended with 10% hydro-processed renewable diesel (HRD) as a carbon source. c. Metabolites in supernatants of cell cultures using 1% HRD as a carbon source . Numbers in b and c indicate fold changes vs control in the order of F50-8, F32-40, and F32-44 (from left to right). Bold font indicates significant abundance changes (*n* = 3, *P* < 0.05).

Extracellular polymeric substances (EPS) are essential for biofilm formation, which depends on nucleotide-diphosphate (NDP) sugar biosynthesis for building block synthesis and assembly.^34,35^ F32-40 and F32-44 possess nearly all the genes required for converting various sugars into NDP sugars, while F50-8 lacks genes for the early biosynthesis steps. All strains have glycosyltransferase (GT) genes responsible for incorporating NDP sugar into EPS, including GT2 (for the assembly of UDP-glucose) and GT4 (for the assembly of GDP-mannose), although the GT4 gene is absent in the strain F32-40. All strains have pilus assembly genes, but only F50-8 has genes for type IV pili (T4P) biosynthesis and assembly, which are crucial for surface sensing and biofilm formation,^36^ underscoring the functional diversity among these strains.

### Fuel-Based Bacterial Growth and Biofilm Formation on CS Coupons

To demonstrate functional diversity among representative strains, we evaluated their fuel-based growth and biofilm formation capabilities as part of the fourth step of the DURABLE workflow. Consistent with the presence of fuel-degrading genes, growth on fuels as carbon sources was indicated by redox indicator color changes (Figure S8b–e) and supported by metabolite analyses of culture supernatants. Of the 263 detected metabolites, four or ten compounds showed significantly altered abundances (*P* < 0.05) compared to the control when using diesel blended with 10% HRD (condition 1, Figure 5b) or HRD (condition 2, Figure 5c), respectively. Under condition 1, key TCA cycle intermediates aconitate and isocitric acid increased in F50-8 and F32-44; 2-furoic acid, a key intermediate in microbial degradation of furanic fuel compounds, increased in F32-44 but declined in F32-40; and (±)­9-HpODE, a linoleic acid (common in biodiesel) degradation product, increased in F50-8. Under condition 2, pyruvic acid, a central metabolite linking multiple pathways, increased in F32-44, while crotonic acid, a fatty acid β-oxidation product, increased in both F50-8 and F32-44. Oleic acid and stearic acid were elevated in three strains, while palmitic acid and myristic acid were elevated in two out of three strains. These changes indicate active metabolism during growth on fuel. Additionally, biofilm formation on CS coupons (Figure S10) correlated with the presence of biofilm-related genes..

### Acidification of the Aqueous Phase and Key Metabolites That Drive or Prevent MIC

Microbial acid production is one of the mechanisms associated with MIC. To assess changes in aqueous-phase pH, the pH of the culture supernatant was measured at the end of the coating period (T) and the exposure period (NF) in R2A medium, and the pH of the aqueous phase was measured at the end of the exposure period under ASW/diesel/air conditions (F) (Figure 6a). At the end of the coating period in R2A, the pH of the corrosive strain F32-44 decreased to ∼5, significantly lower (*P* < 0.001) than that of the non-corrosive strains (F50-8 and F32-40) and the R2A control (∼8.2). This trend persisted throughout the exposure period in R2A. Under ASW/diesel/air conditions, all three cultures reached a similar acidic pH (∼6, *P* > 0.5), whereas no comparable pH decrease was observed in the R2A control, consistent with previous studies of fungal isolates from fuel storage tanks under ASW/B20 conditions.^16^ The prominent acidification of F32-44 suggested organic acid production that likely promoted MIC by lowering local pH and accelerating metal dissolution.

**Figure 6:**
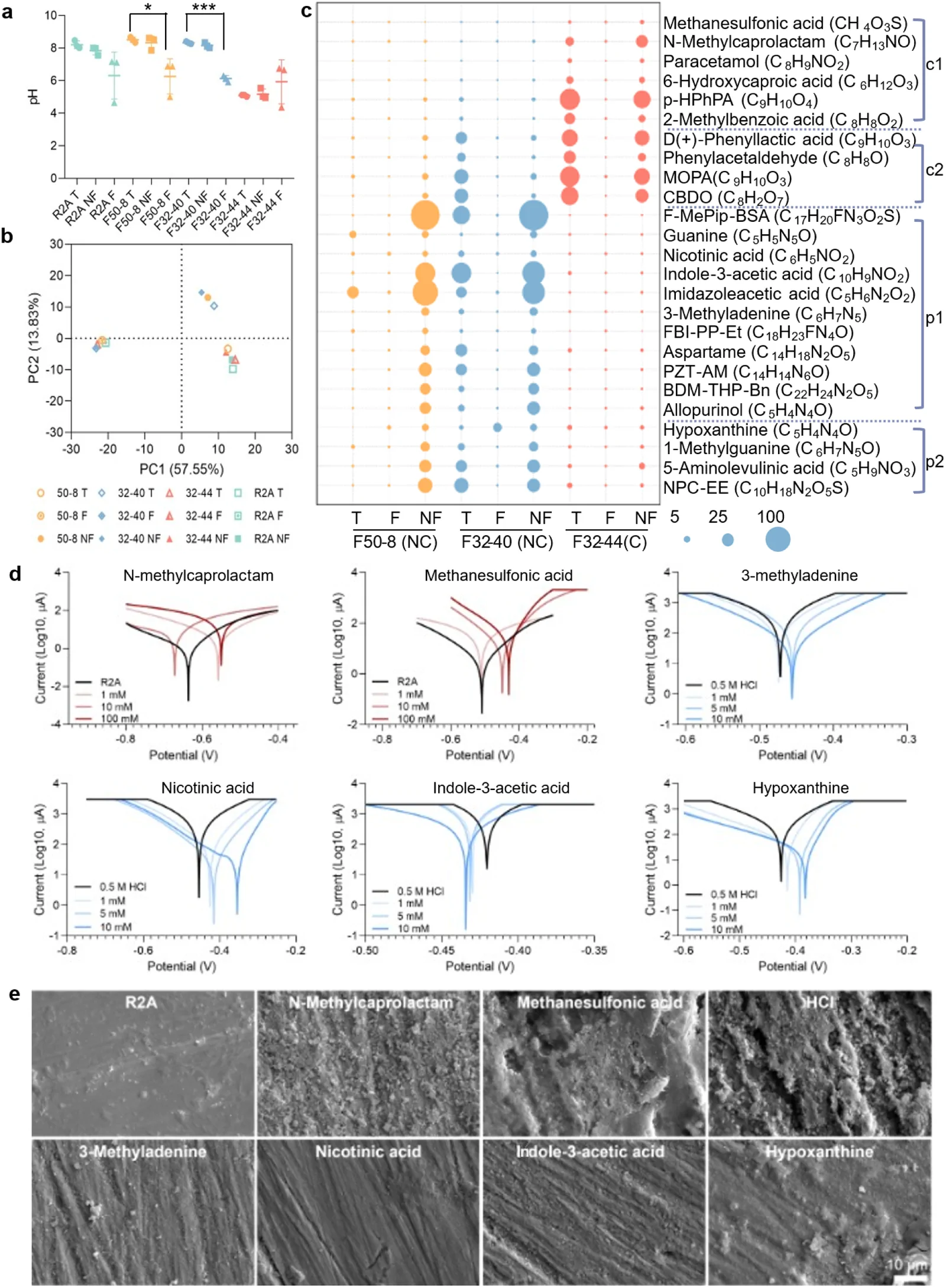
pH value changes over time and key metabolites associated with MIC in the representative bacterial strains. a. pH values of the supernatant at the end of incubation in R2A for 9 days (T) and at the end of exposure in R2A (NF) or ASW/fuel/air phase (F) for 25 days. *, *P* < 0.05; ***, *P* < 0.001. b. Overall similarities of metabolite profiles in cell culture supernatants after incubation in R2A for 9 days (T, blank symbol) and exposure to ASW/fuel/air (F, pattern fill), or R2A for 25 days (NF, filled symbol). c. Fold changes of the metabolites significantly altered compared to the R2A control group. These include metabolites that significantly increased in the corrosive strain F32-44 only (c1), noncorrosive strains only (p1), or in both types of strains but were significantly higher in the corrosive strain F32-44 (c2) or noncorrosive strains (p2). C, corrosive; NC, noncorrosive. d. Electrochemical validation of a few key metabolites on CS corrosion tests. Nyquist plots after incubation in R2A or 0.5 M HCl for 24 h with different concentrations of specific metabolites at room temperature. e. Surface morphology of the metal coupons after 24 h exposure to two corrosive metabolites or four protective metabolites (10 mM), as well as controls (0.5 M HCl or R2A). Scale bar: 10 μm. Abbreviations in b: p-HPhPA, DL-4-Hydroxyphenyllactic acid; MOPA, DL-α-Methoxyphenylacetic acid; CBDO, 3-Hydroxy-4-(2-hydroxy-3,4-dioxocyclobuten-1-yl)­oxycyclobut-3-ene-1,2-dione; F-MePip-BSA, 4-fluoro-N-[4-(4-methylpiperazino)­phenyl]­benzenesulfonamide; FBI-PP-Et, 1-{4-[(3S)-3-(5-Fluoro-1H-benzimidazol-2-yl)-1-pyrrolidinyl]-1-piperidinyl}­ethenone; PZT-AM, N1-[4-(3-pyrazin-2-yl-4,5-dihydro-1H-1,2,4-triazol-5-yl)­phenyl]­acetamide; BDM-THP-Bn, benzyl 4-{[(1,3-benzodioxol-5-ylmethyl)­amino]­carbonyl}­tetrahydro-1­(2H)-pyridinecarboxylate; NPC-EE, N-(3-nitratopivaloyl)­cysteine ethyl ester.

To identify the key metabolites that drive or inhibit MIC, we conducted a comparative metabolomic analysis of three phylogenetically and functionally distinct strains in the fifth step of the DURABLE Workflow. Metabolite profiles from bacterial culture supernatants were compared before (after 9 days in R2A) and after 25 days of exposure to different environments (ASW/fuel/air phase or R2A) (Figure S7). A total of 477 metabolites were detected (Figure S7). Metabolites were classified by accumulation patterns (*P* < 0.05): (c1) corrosive strain only; (c2) both strain types but higher in the corrosive strain; (p1) noncorrosive strain only; (p2) both strain types but higher in the noncorrosive strain. Groups c1 and c2 may drive MIC, while groups p1 and p2 may offer protection against MIC.

Metabolite profiles showed clear differences between the corrosive strain and noncorrosive strains, between the two noncorrosive strains, and between the start and end of the incubation periods (Figure 6b). The numbers of metabolites in groups c1 to p2 were 6, 4, 11, and 4, respectively (Figure 6c). Among the three c1 metabolites that accumulated over time in F32-44, methanesulfonic acid (MSA) is a known strong organic acid with documented corrosivity at high temperature (95 °C) and concentration (25% in HCl),^37^ or at 70% in water.^38^ *N*-methylcaprolactam has no known linkage to corrosion, while paracetamol (acetaminophen) has been reported as a copper corrosion inhibitor.^39^ The other three c1 metabolites decreased by the end of exposure; although 2-methylbenzoic acid has not been studied, benzoic acid derivatives are known to have anticorrosion effects.^40^ Of the four c2 metabolites, only phenylacetaldehyde has reported corrosion-inhibiting properties.^41^ Most p1 and p2 metabolites were nitrogen-containing heterocycles with functional groups such as −NH_2_, alkyl chains, or −CH_2_COOH (Figure S11), which are known for strong metal adsorption and corrosion inhibition.^42^ These results suggest that the corrosion phenotype was determined by a mix of corrosive and protective metabolites.

### Validation of Corrosive or Protective Effects of Key Metabolites

To validate the corrosive or protective effects of key metabolites, electrochemical analysis using standard compounds was conducted in the fifth step of the DURABLE workflow (Figure 6d). Two c1 metabolites, MSA and *N*-methylcaprolactam, showed concentration-dependent increases in corrosion current density (I_corr_) in R2A medium, consistently exceeding the control, indicating their roles as corrosion accelerators. In contrast, three p1 and one p2 metabolites reduced both cathodic and anodic I_corr_ in 0.5 M HCl with increasing concentration, consistent with the increased surface coverage (η), indicating their effectiveness as potential corrosion inhibitors under acidic conditions (Table S2).

Scanning electron microscopy (SEM) analyses of carbon steel surfaces after 24 h immersion (Figure 6e) showed smooth surfaces with minor microscratches in R2A. In contrast, exposure to MSA or *N*-methylcaprolactam resulted in pronounced surface roughness and deposits, indicating corrosion. Severe degradation was observed in 0.5 M HCl, but the addition of 3-methyladenine, nicotinic acid, indole-3-acetic acid, or hypoxanthine significantly reduced surface damage, confirming their protective effects.

## Discussion

In this study, we developed DURABLE, a systematic pipeline for rapid bacteria isolation from fuel tank biofilms and corrosion phenotype screening, as well as the identification of key metabolites associated with MIC. Microbial communities from diesel and HRD fuel tanks were profiled at higher sequencing depth (more than 32K 16S rRNA and more than 12K ITS reads per sample). Novel MIC-associated bacterial strains were isolated via an agarose gel droplet microfluidics system, and a high-throughput 96-well plate assay utilizing CS beads enabled rapid screening of corrosion phenotypes. MIC-associated metabolites were identified via analysis of the representative corrosive or noncorrosive strains and validated through electrochemical testing.

Previous studies have reported conflicting findings on whether the fuel type influences microbial community composition. In this study, we observed that the microbial community in fuel storage tanks varies by both fuel type and spatial scale. Although bacterial richness in both tanks was comparable to that reported in the car tank lid microbiome,^10^ oil storage tanks,^20^ and ethanol-contaminated gasoline tanks,^8^ both bacterial and fungal richness were much lower than those observed in B20 storage tanks,^9^ implying a more complex relationship between fuel type and microbial diversity. In addition to the community profiling, diverse bacteria were isolated and characterized at a single-strain resolution. Among three representative MIC-associated strains studied here, *Microbacterium* and *Cellulosimicrobium* strains were isolated directly from fuel storage tanks for the first time, although both strains were isolated from the enrichment cultures of car tank lid microbiomes.^10^ A *C. cellulans* strain isolated from a tarball was capable of degrading alkane.^43^ This study also presents the first isolation of an *A. lwofii* strain from a fuel tank, supporting earlier 16S rRNA findings of *A. sp.* in diesel storage tanks^7^ and oil-degrading communities.^44^ Other *A. lwofii* strains have also been isolated from crude oil-contaminated soil.^45,46^ Given that both *Acinetobacter* and *Cellulosimicrobium* were below the detection limit in the 16S rRNA data, the recovery of these strains demonstrates the effectiveness of the agarose gel droplet microfluidic system used here in isolating bacterial strains from microbial communities.^17^ However, overall cultivable bacterial diversity was not assessed due to the limited number of isolates (<100) tested.

The combination of a rapid, high-throughput 96-well plate CS bead assay with metabolite profiling offers a powerful strategy for rapidly identifying MIC-associated metabolites. The 96-well format greatly improved the screening efficiency, overcoming a major bottleneck in analyzing MIC-associated microorganisms and enabling more effective downstream mechanistic analysis. Comparative profiling of bacterial culture supernatants across conditions and time points revealed both corrosive and protective metabolites. Notably, the protective compound hypoxanthine also accumulated in the corrosive strain F32-44, though at lower levels than in noncorrosive strains, suggesting that overall corrosivity is shaped by the dominant metabolite profile rather than the presence of individual compounds. These findings support the dual role of microorganisms in promoting or inhibiting corrosion^47^ and provide direct evidence of the complex nature of MIC. The identified noncorrosive strains and protective metabolites offer potential for MIC mitigation in fuel tanks. Future studies using microbial consortia derived from the isolated microbial strains, especially bacteria-fungus consortia, will help further unravel MIC mechanisms and support the development of targeted corrosion control strategies.

Well-known MIC-associated microorganisms, particularly sulfate-reducing bacteria, were not recovered using DURABLE. One possible explanation is that prolonged storage of biofilm samples under oxic conditions prior to initiating the DURABLE workflow may have compromised the viability of strict anaerobes. Conducting sample collection, storage, and processing (e.g., gel droplet generation and incubation) and CS bead-based phenotypic screening under anoxic conditions may enable DURABLE to recover and identify MIC-associated strict anaerobes, as the screening and characterization methods are based on measuring the end outcome—corrosion—regardless of the mechanisms of corrosion. Moreover, the identified corrosion-causing strains likely represent primary corrosion-causing microorganisms in this sample, as acid-producing bacteria are widely associated with corrosion in fuel pipeline and storage systems through organic acid production (13).

## Materials and Methods

### Biological Samples

Six samples were collected from different locations (tank side, interface, or floating mat) within two fuel storage tanks in July 2023 (Figures 1, Figure S1). Fuels were obtained through the fuels group at the Naval Air Warfare Center Aircraft Division in Patuxent River, MD. The fuels included conventional petroleum-based F76 Navy diesel (F76) and algal hydroprocessed renewable diesel (HRD), both qualified to F76 specifications (MIL-DTL-16884). In comparison to commercial diesel, F76 has required additives, including a metal deactivator and lubricity improver, as well as a maximum fatty acid methyl ester (FAME biodiesel) content of 0.1% by volume. The fuels were not chemically characterized. The fuel tanks were black high-density polyethylene mock fuel tanks. Rainwater contaminated the tanks during storage at the U.S. Naval Research Laboratory (Stennis Space Center, MS), which resembles realistic outdoor fuel storage tank situations. The F76 fuel was light orange with a gray mass (samples F25 and F26) at the fuel/water interface (Figure S1). Sample F32 consisted of one large dark-brown mat at the fuel/water interface. The HRD fuel was very pale yellow, with patches of dark and white material (samples F9 and F10) at the fuel/water interface (Figure S1). Sample F50 was a gelatinous black material on the side walls of the HRD tank fuel layer and was collected using sterile cotton swabs. All other fuel/water interface samples were collected using a sterile pipet/hose/bulb setup with a sterile 50 mL pipet connected to PVC tubing. The contents were placed onto a 0.22 μm hydrophilic filter, allowing the water phase to pass through. The fuel layer was decanted using a sterile pipet. The filters were quartered using sterile scissors and placed into separate lysing tubes. Only the floating mat (sample F32) from the F76 fuel tank interface was pulled into the pipet and then placed into a 2 mL centrifuge tube. The samples were stored at −80 °C before analysis through the DURABLE workflow.

### 16S rRNA and ITS Amplicon Sequencing

DNA was isolated from all samples using the FastDNA Spin Kit for Soil (MP Biomedicals, 116560300), following the manufacturer’s manual. 16S rRNA and ITS amplicon sequencing were conducted at Zymo Research (Irvine, California). Briefly, the amplicon libraries were prepared using the Quick-16S Plus NGS Library Prep Kit and the primer sets Quick-16S Primer Set V3–V4 targeting 16S rRNA and ITS2 Primer Set targeting ITS (Zymo Research). Sequencing was conducted on the Illumina NextSeq 2000 with the P1 reagent kit (600 cycles) with a 30% PhiX spike-in. The Dada2 pipeline^48^ was used to remove chimeric sequences and infer the unique amplicon sequences from the raw reads. Uclust from Qiime v.1.9.1 was used for taxonomy assignment using a database containing references for 16S, ITS, and 18S that is internally designed and curated at the Zymo Research Database. Composition visualization, alpha-diversity, and beta-diversity analyses were performed with Qiime v.1.9.1.^49^

### Single-Cell Gel Droplet Encapsulation and Culture for Bacterial Strain Isolation

Prior to single-cell encapsulated droplet generation, samples F50 (HRD tank) and F32 (F76 tank) were stained using the LIVE/DEAD *Bac*Light Bacterial Viability Kit (Cat. No. L7007) for cell viability assessment and FilmTracer SYPRO Ruby Biofilm Matrix Stain (Cat. No. F10318, ThermoFisher) for biofilm staining. The excitation and emission wavelengths were 495/518 nm for green fluorescence, 514/615 nm for PI, and 450/610 nm for Ruby.

Single-cell agarose gel droplet encapsulation was performed to isolate bacterial strains using a protocol similar to a recently developed droplet microfluidics method for improved preservation and isolation of environmental microorganisms.^50^ Briefly, samples F50 and F32 were vortexed in R2A medium to dissociate biofilm aggregates and suspend the microbial cells. About 1 million cells were mixed with 1% (w/v) low-melting-point agarose prepared using R2A and encapsulated into water-in-oil agarose microdroplets (∼100 μm in diameter) using a flow-focusing droplet microfluidic device with HFE-7500 fluorinated oil containing 2% PicoSurf as the continuous phase. All tubing and channels were preheated to 30 °C to prevent premature gelation. After generation, droplets were stored at 4 °C for 10 min to allow agarose gelation. Then, 10% (v/v) perfluoro-1-octanol (PFO) in HFE-7500 was added for demulsification, and the gel droplets were separated from the oil phase by centrifugation at 2,000 rpm. The cleaned gel droplets were then incubated in R2A medium in 6-well plates at 30 °C for 7 days with daily medium replacement. Agarose gel droplets were then washed thoroughly using a 20 μm cell strainer to remove ambient bacteria not encapsulated within the gel droplets. The cleaned agarose gel droplets were then re-encapsulated into R2A-containing water-in-oil emulsion droplets using a double-emulsion protocol to create a protective barrier to prevent bacterial escape from the gel during droplet dispensing. Finally, the double-emulsion droplets were dispensed onto R2A agar plates at ∼ 200 droplets per plate at single-droplet (i.e., single clonal cell) resolution. Plates were incubated at 30 °C for 72 h to allow colony formation. Bacterial colonies were picked and streaked on R2A plates to obtain pure bacterial clones.

### Metal Corrosion Phenotype Test Using Carbon Steel (CS) Beads in a 96-Well Plate

Carbon steel beads (500 μm, MSE Supplies) were sterilized by autoclaving at 121 °C for 15 min before being added to each well of a 96-well plate. CS beads covered about half of the bottom surface area to allow for easy corrosion phenotype observation under a microscope. The starting OD_600_ of the bacterial cell culture was about 0.1, prepared by diluting a 24-h revived culture into fresh R2A medium. The 24-h revived culture was initiated from glycerol stocks (1:100 inoculum) and had reached the stationary phase. R2A medium alone was used as the negative control. The sample volume per well was 150 μL. Plates were incubated at 30 °C, and microscopy images were taken at 40× magnification.

### Metal Corrosion Test Using CS Coupons under Different Conditions

Carbon steel shim coil (0.002” thickness, Maudlin Products, S005-6-100) was cut into 1 cm × 4 cm coupons and sterilized using acetone and ethanol. The coupons were air-dried in a biosafety cabinet, followed by exposure to UV light for 15 min on each side. The initial weight of each coupon was measured to four decimal places before being placed into a glass vial (30 mL, Uline, S-25277C) for the metal coupon weight loss test.

The bacterial cell cultures were revived from glycerol stocks using R2A for 24 h at 30 °C with shaking (200 rpm). Cell culture (15 mL, OD_600_ = 0.1) was added into each glass vial containing a CS coupon, where R2A was used as a negative control, with seven replicates each. All test samples were kept at 30 °C without shaking. On day 4, 1 mL of cell culture in each vial was exchanged with 1 mL of fresh sterile R2A medium. On day 9, CS coupons in 3 replicates were transferred to new glass vials containing 3 mL of artificial sump water (ASW) without a carbon source^16^ and 9 mL of diesel, designated as ASW/fuel/air phases, mimicking real-world fuel tank conditions (Figure S5). CS coupons in 3 replicates were kept in the R2A condition. CS coupons in one replicate were used for biofilm staining using FilmTracer SYPRO Ruby Biofilm Matrix Stain.

At the end of the incubation period (25 days), the coupons were cleaned following the ASTM G1-03 standard. Briefly, CS coupons were placed in 1.8 M HCl containing 2% propargyl alcohol at 70 °C for 1 min, then immersed in saturated calcium hydroxide solution at room temperature for 30 s to neutralize, scrubbed on both sides using a non-scratching nylon brush, and rinsed in deionized water. These steps were repeated 3 times. Finally, the coupons were rinsed with acetone, air-dried, and the weight of each coupon was measured. The average corrosion rate (CR, mpy) was calculated using the formula CR = (144 × W)/(A × T × D), where W is weight loss (mg), A is exposure surface area (cm^2^, 8 for both sides of 1 × 4 cm coupon), T is time of exposure (day, 34 days), and D is density (g/cm^3^, 7.874). Iron concentrations in the supernatants were measured using an iron assay kit (Sigma-Aldrich, MAK025) following the manufacturer’s instructions. Supernatants were collected by centrifuging 1 mL of cell cultures at 12,500 rpm for 5 min, followed by filtering through a 0.22 μm filter, and then stored at −80 °C for metabolite analyses.

### Whole Genome Sequencing and Analysis of the Representative Bacterial Strains

Bacterial DNA was isolated using an E.Z.N.A. Bacterial DNA Kit (Omega BIO-TEK, D3350-02). Whole genome sequencing was conducted at Plasmidsaurus (Arcadia, CA 91006, USA) using Oxford Nanopore Technology with custom analysis and annotation. Full-length 16S rRNA sequences of the representative strains F50-8, F32-40, and F32-44 were first blasted against the standard nr database in NCBI to determine the taxonomies and the most similar 16S rRNA sequences. Subsequently, full-length 16S rRNA sequences of the selected reference genomes were obtained from the NCBI database and used to construct full-length 16S rRNA sequence-based phylogenetic trees in MEGA 11 using the neighbor-joining tree method with 1000 bootstrap replicates.^51^

### Bacterial Growth Using Fuel as a Carbon Source

Bacterial cell cultures were revived in R2A for 24 h, followed by three washing steps using carbon-free Bushnell Haas broth medium (BH, Sigma-Aldrich, B4926). The washed cells were resuspended in BH and adjusted to OD_600_ = 0.1 for the growth test. Filter-sterilized HRD fuel or HRD-blended diesel fuel (10% HRD) was added to BH as the carbon source (1% v/v). Tween 80 (0.1% v/v) and the redox indicator 2,6-dichlorophenol indophenol (DCPIP, 0.05 mg/mL) were also supplemented in the growth medium.^52^ After 7 days of culture at 30 °C with shaking (200 rpm), 1.5 mL of cell cultures were centrifuged for 5 min at 10,000 × g, and the supernatants were filtered using a 0.22 μm filter and stored at −80 °C for metabolite assays .

### Analysis of the Metabolites in the Supernatants

Supernatants (50 μL) were extracted using 300 μL of methanol, and untargeted liquid chromatography high-resolution accurate mass spectrometry (LC-HRAM)^53^ was carried out at the Texas A&M University Integrated Metabolomics Analysis Core (IMAC) (College Station, TX, USA). Briefly, LC-HRAM was performed in negative ionization mode on a QExactive Plus Orbitrap mass spectrometer (Thermo Scientific, Waltham, MA, USA) coupled to a binary pump HPLC (UltiMate 3000, Thermo Scientific). Full MS data were obtained at resolutions of 35,000 (MS1) and 17,500 (MS2) with a 1.5 *m*/*z* isolation window and a stepped NCE (20, 40, 60). A Kinetex 2.6 μm 100 × 2.1 mm Polar C18 column (Phenomenex, Torrance, CA, USA) was used for chromatographic separation at 30 °C. A reverse-phase solvent gradient method using solvent A (5 mM ammonium formate in water) and solvent B (methanol) was applied. Xcalibur (Thermo Scientific) was used for data processing. Pooled samples were used as quality controls (QCs), spaced at one QC per 15 samples. Compound Discoverer v3.0 was used to annotate the metabolite features against the mzCloud and ChemSpider databases.

### Validation of the Effects of Metabolites on CS Corrosion

Reference chemicals were purchased from Sigma-Aldrich. The potential corrosive compounds, methanesulfonic acid and *N*-methylcaprolactam were dissolved in R2A medium at concentrations of 1, 10, and 100 mM. The potential protective compounds, 3-methyladenine, nicotinic acid, indole-3-acetic acid, and hypoxanthine, were dissolved in a 0.5 M HCl solution at concentrations of 1, 5, and 10 mM. Electrochemical experiments were conducted using a MultiPalmSens4 potentiostat. The working electrode was carbon steel 1010 (Maudlin) with an exposed area of 3 cm^2^. Prior to testing, the steel surface was polished sequentially with sandpaper of 400, 1000, 1500, and 2000 grid, and rinsed thoroughly with distilled water following ASTM G1-03 protocol. Electrode potentials were measured against an Ag/AgCl reference electrode (Gamry), and a carbon rod electrode (Gamry) was used as the counter electrode. The corrosion potential (E_corr_) and linear polarization curves were recorded following the immersion of the working electrode in the test solutions for 24 h. Potentiodynamic polarization curves were obtained by scanning the potential range from −200 mV + E_corr_ to 200 mV + E_corr_ at a scan rate of 10 mV/s with a 0.5 mV step voltage. Surface coverage value was calculated using the equation $\eta =\frac{I_{blank}-I_{sample}}{I_{blank}}$ . Surface morphology of the carbon steel was assessed using an FEI Helios NanoLab 460F1 FIB-SEM system.

## Supplementary Material



## Data Availability

The raw data of 16S rRNA and ITS amplicon sequencing have been deposited into the NCBI BioProject database under accession number PRJNA1344803. The whole genome sequences of the bacterial strains have been deposited in NCBI under BioSample accessions SAMN52654226 (F50–8), SAMN52654227 (F32-40), SAMN52654228 (F32-44), SAMN56688088 (F50-1), SAMN56688089 (F50-5), SAMN56688090 (F50-27), SAMN56688091 (F32-32), SAMN56688092 (F32-37), SAMN56688093 (F50-35), SAMN56688094 (F32-3), and SAMN56688095 (F32-42).

## Notes

**Figure S1. Images and information on microbial samples collected from the two fuel storage tanks. a.** Images of fuel/water samples from F76 and HRD tanks. Sample tubes are 19 mm in diameter. **b.** Images of the samples analyzed in this study. **c.** Detailed information on the samples analyzed in this study. **Figure S2. Rarefaction cures of the amplicon sequencing data. a.** 16S rRNA sequencing data. **b.** ITS sequencing data. **Figure S3. Richness and Shannon index as well as Bray-curtis dissimilarities between samples from the HRD and F76 diesel tanks**. **a**–**c.** Bacterial communities. **d**–**f.** Fungal communities. Blue font indicates samples from the HRD tank, and red font indicates samples from the diesel tank. HRD: hydro-processed renewable diesel; F76: petroleum-based Navy F76 diesel. **Figure S4. Live/dead and biofilm matrix fluorescence staining of sample F50. a.** Live cells stained with SYTO9, shown in the green fluorescence channel. **b.** Dead cells stained with propidium iodide, shown in the red fluorescence channel. **c.** Biofilm matrices stained with FilmTracer SYPRO Ruby, represented in blue for visual differentiation from the red signal. **d.** Merged overlay of all three channels. **Figure S5. Bacteria recovery using single-cell gel droplet encapsulation and culture-based microfluidics technology. a.** Single microbial cells from sample F32 (F76 tank) were encapsulated into gel droplets. Red arrows indicate fluorescently stained bacteria in a gel droplet. Scale bar: 100 μm. **b.** Proliferated microbial cells in gel droplets after 48 h of incubation. Scale bar: 20 μm. **c.** Bacterial clones formed on LB plates after 72 h of incubation, following the dispensing of cell-encapsulated gel droplets onto plates. **Figure S6. Images of metal corrosion phenotype screening using CS beads in 96-well plates.** In total, 88 bacterial isolates from two biofilm samples, F50 and F32, were screened for their corrosivity. **a**. Time 0. **b**. After 2 days of incubation. **c**. After 7 days of incubation. **d**. Plate layout. Blue highlight: bacterial isolates from sample F50. Orange highlight: bacteria isolated from sample F32. NC: negative control (R2A only, without bacteria). Bordered strains represent those with a distinct corrosion phenotype and sequenced genomes. Blue circles indicate noncorrosive strains, and red circles indicate corrosive strains **Figure S7. Carbon steel metal coupon corrosion weight loss experiment and metabolite assay. a.** Experiment flowchart. **b.** Heatmap generated by hierarchical analysis of the metabolite profiles at the beginning and end of the experiment. Suffix T indicates the end of incubation in R2A and before being exposed to different environments; suffix F or NF indicates the end of exposure to ASW/fuel/air conditions or R2A, respectively. N indicates negative control (R2A). Log_10_ transformed data were used for analysis. The red frame indicates metabolites only accumulated in the corrosive strain F32-44, and the blue frame indicates metabolites only accumulated in noncorrosive strains F50-8 and F32-40. **Figure S8. Bacteria strain morphologies and growth on fuel. a**. Bacterial morphologies on R2A plates. Color changes after 36 h (**b, c**) or 84 h (**d, e**). Cells were incubated in carbon-free Bushnell Haas broth medium containing 1% HRD (**b, d**) or 1% HRD-blended diesel (diesel: HRD = 9:1) (**c, e**) as the carbon source, with redox indicator 2,6-dichlorophenolindophenol (DCPIP, 0.05 mg/mL). **Figure S9. Summary of the relative abundance of the top 20 most abundant genera across three sets of samples collected at different times from two tanks (HRD tank and F76 diesel tank) and the genera from which bacterial isolates were obtained.** *Column: sample batch number from which bacterial strains were isolated and sequenced. Each heatmap column represents one sample. Sample batch 1 was analyzed in this study, whereas batches 2 and 3 were collected later from the same tanks. E indicates bacterial strains were isolated from enrichment cultures. **Figure S10. Biofilms on the CS coupons stained with fluorescence dye Ruby after 9 days of incubation in R2A.** Biofilm matrices stained with FilmTracer SYPRO Ruby. C, corrosive strain; NC, noncorrosive strain. Scale bar: 100 μm. **Figure S11. Molecular structures of the key metabolites accumulated in the bacteria culture supernatants. a.** Metabolites specifically accumulated in corrosive strain F32–44. **b.** Metabolites accumulated in all strains but with higher abundances in corrosive strain F32–44. **c**. Metabolites specifically accumulated in noncorrosive strains F50-8 and F32-40. **d.** Metabolites accumulated in all strains but with higher abundances in noncorrosive strains. **Table S1.** Genome sequence information of the isolated strains. **Table S2.** Potentiodynamic polarization parameters of CS coupons immersed in various solutions at room temperature for 24 h (PDF)
