# Supplementary material for: DURABLE: A Workflow for Determining Corrosion-Driving and Protective Microbial Mechanisms
Source: Environ Sci Technol. 2026 Aug 11;60(33):23319–32. doi: 10.1021/acs.est.6c07071 (PMC13523691; doi:10.1021/acs.est.6c07071)
Supplement: Supplementary file 1 [file es6c07071_si_001.pdf]

Supplementary information for "DURABLE: A Workflow for Determining Corrosion-Driving and Protective Microbial Mechanisms"

Aifen Zhou[a§], Yuwen Li[b§], Han Zhang[b], Evelyn Callaway[a], Song-I Han[b], Jason S. Lee[c], Kyongbum Lee[d], Arul Jayaraman[a,e], Paul de Figueiredo[f,g,h*], Arum Han[a,b,e*]

[a]Department of Chemical Engineering, Texas A&M University, College Station, TX 77843, USA

[b]Department of Electrical and Computer Engineering, Texas A&M University, College Station, TX 77843, USA

[c]U.S. Naval Research Laboratory, Stennis Space Center, Mississippi, MS39529, USA

[d]Department of Chemical and Biological Engineering, Tufts University, Medford, MA 02155, USA

[e]Department of Biomedical Engineering, Texas A&M University, College Station, TX 77843, USA

[f]Christopher S Bond Life Sciences Center, University of Missouri, Columbia, MO 65211, USA

[g]Department of Molecular Microbiology and Immunology, University of Missouri School of Medicine, Columbia, MO 65211, USA

[h]Department of Veterinary Pathobiology, University of Missouri School of Veterinary Medicine, Columbia, MO 65211, USA

§A.Z. and Y.L. contributed equally to this work

* Email: Paul de Figueiredo, paullifescience@health.missouri.edu

* Email: Arum Han, arum.han@ece.tamu.edu

Number of pages: 14

2 Tables and 10 Figures

Supplementary Tables

Table S1. Genome sequence information of the isolated strains

Strain ID	F50-1	F50-5	F50-27	F32-32	**F32-44**	F50-35	F32-3	F32-37	**F32-40**	F32-42	**F50-8**
Genome size (bp)	4,333,526	4,333,880	4,333,616	4,333,577	**4,333,845**	3,569,186	3,629,899	3,674,282	**3,560,658**	3,553,728	**3,723,910**
GC content (%)	74.5	74.5	74.5	74.5	**74.5**	70.3	70.0	70.0	**70.1**	70.1	**42.7**
Genes annotated	3,892	3,946	3,902	3,911	**3,935**	3,449	3,530	3,587	**3,447**	3,442	**3,723**
CDS	3,826	3,880	3,836	3,845	**3,869**	3,394	3,476	3,532	**3,392**	3,387	**3,603**
tRNA	52	52	52	52	**52**	46	46	46	**46**	46	**87**
rRNA	9	9	9	9	**9**	6	6	6	**6**	6	**21**
ncRNA	4	4	4	4	**4**	2	2	2	**2**	2	**11**
tmRNA	1	1	1	1	**1**	1	1	1	**1**	1	**1**
Number of reads	132,194	18,073	35,791	102,634	**20,688**	58,586	53,004	536,034	**702,510**	49,862	**65,559**
Longest read (bp)	109,247	102,799	106,115	85,586	**91,345**	97,280	89,503	78,337	**98,445**	63,735	**104,760**
Assembled coverage (×)	99	33	67	101	**39**	78	80	92	**95**	37	**59**
Number of contig	1	1	1	1	**1**	4	3	4	**3**	4	**10**
Completeness	100.0%	96.4%	98.0%	99.7%	**98.0%**	99.5%	99.5%	99.5%	**99.5%**	99.5%	**99.8%**
Closest reference species	*Cc*	*Cc*	*Cc*	*Cc*	***Cc***	*M*	*M*	*M*	***M***	*M*	***Al***
NCBI accession number	SAMN56688088	SAMN56688089	SAMN56688090	SAMN56688091	SAMN52654228	SAMN56688093	SAMN56688094	SAMN56688092	SAMN52654227	SAMN56688095	SAMN52654226

Bold font indicates representative strains; CDS: coding DNA sequence; ncRNA: non-coding RNA; tmRNA: transfer-messenger RNA
Cc: Cellulosimicrobium cellulans; M: Microbacterium sp.; Al: Acinetobacter lwoffii

Table S2. Potentiodynamic polarization parameters of CS coupons immersed in various solutions at room temperature for 24 h.

	Concentration (mM)	E_{corr} (mV vs. Ag/AgCl)	I_{corr} (μA/cm2)	η
N-Methylcaprolactam	R2A	-638	5	
	1	-553	12	-1.40
	10	-677	22	-3.40
	100	-558	29	-4.80
Methanesulfoinc acid	R2A	-508	4	
	1	-513	17	-3.25
	10	-448	42	-9.50
	100	-435	120	-29.00
3-Methyladenine	0.5 M HCl	-474	300	
	1	-466	185	0.38
	5	-456	51	0.83
	10	-456	42	0.86
Nicotinic acid	0.5 M HCl	-455	218	
	1	-426	58	0.73
	5	-416	22	0.90
	10	-354	44	0.80
Idole-3-acetic acid	0.5 M HCl	-420	302	
	1	-431	50	0.83
	5	-430	41	0.86
	10	-434	29	0.90
Hypoxanthine	0.5 M HCl	-424	250	
	1	-407	129	0.48
	5	-392	71	0.72
	10	-387	117	0.53

Supplementary Figures

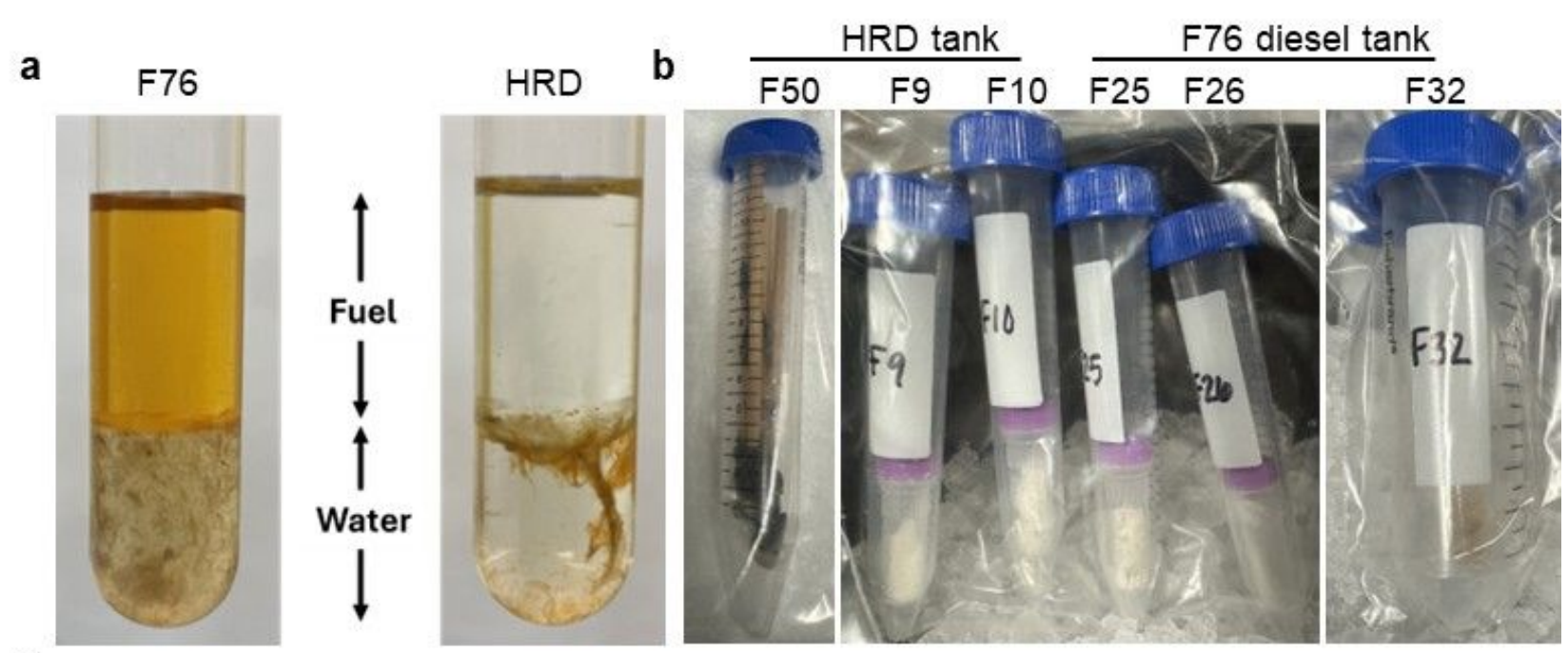

c

Sample ID	Fuel type	Sample location	Sample method	Volume filtered (ml)	Collection vial
F50	HRD	Tank side wall	Sterile cotton swabs (triplicate)	N/A	Falcon tube, 15 ml
F9	HRD	Fuel/water Interface	Sterile pipet (50 ml) / 0.22 μm filter	130	Lysing matrix tube
F10	HRD	Fuel/water Interface	Sterile pipet (50 ml) / 0.22 μm filter	130	Lysing matrix tube
F25	F76 diesel	Fuel/water Interface	Sterile pipet (50 ml) / 0.22 μm filter	208	Lysing matrix tube
F26	F76 diesel	Fuel/water Interface	Sterile pipet (50 ml) / 0.22 μm filter	208	Lysing matrix tube
F32	F76 diesel	Floating mat	Sterile pipet (50 ml) / 0.22 μm filter	N/A	Centrifuge tube, 2ml

Fig. S1. Images and information of microbial samples collected from the two fuel storage tanks. a. Images of fuel/water samples from F76 and HRD tanks. Sample tubes are 19 mm in diameter. **b.** Images of the samples analyzed in this study. **c.** Detailed information of the samples analyzed in this study.

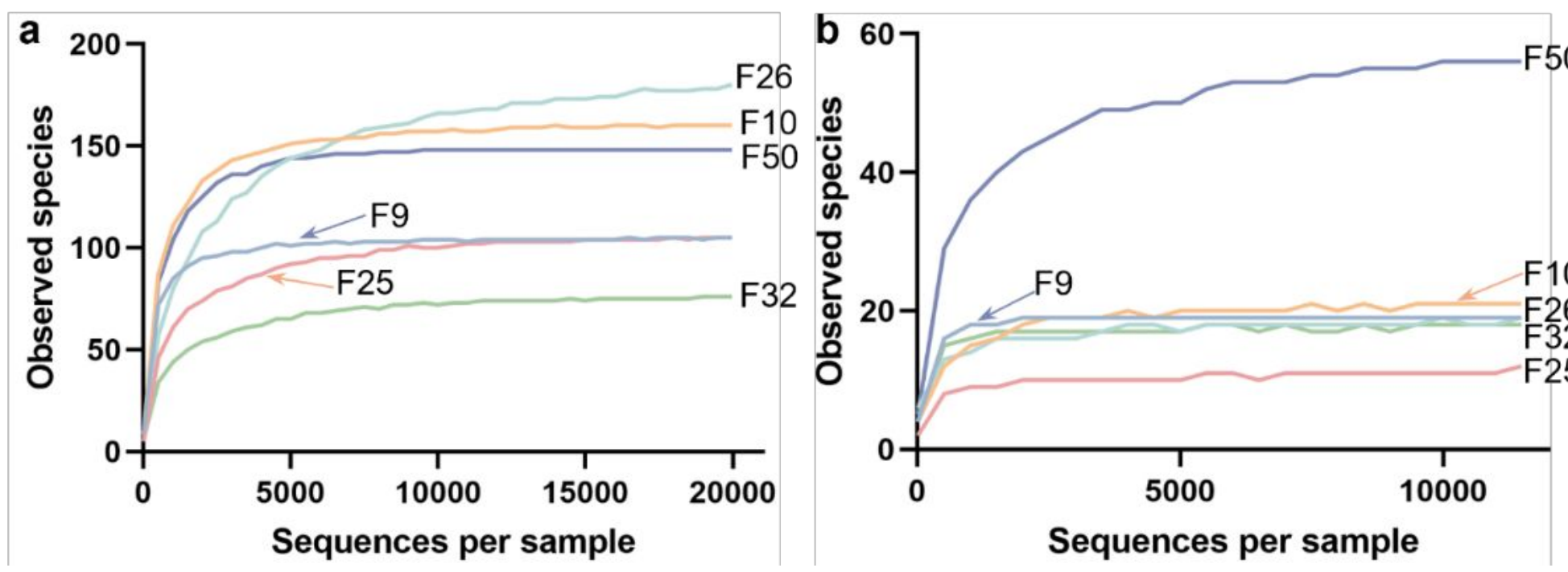

Fig. S2. Rarefaction cures of the amplicon sequencing data. a. 16S rRNA sequencing data. **b.** ITS sequencing data.

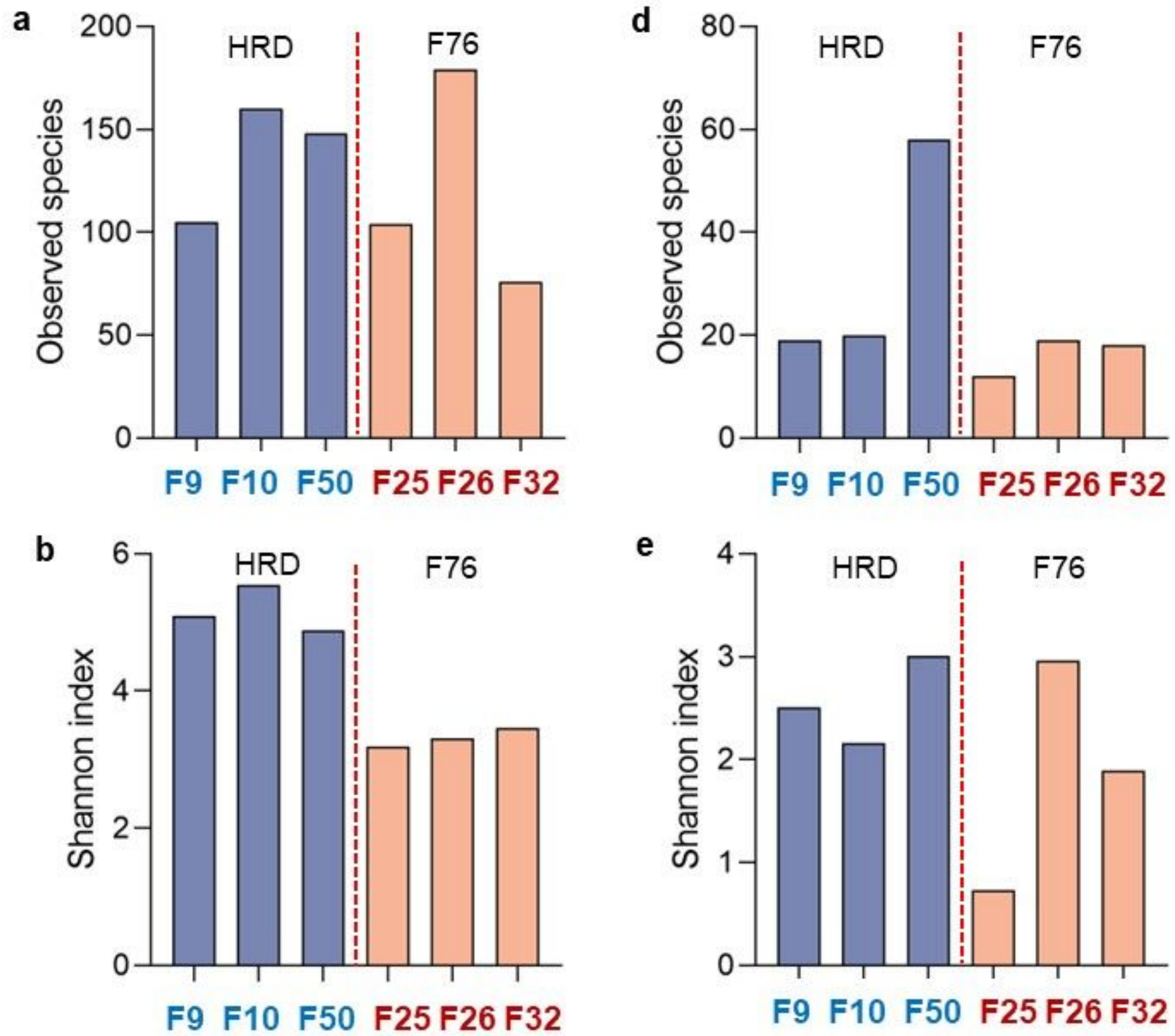

c The Bray-Curtis dissimilarities among samples

	F9	F10	F50	F25	F26	F32
F9	0.00	0.25	0.57	0.83	0.83	0.93
F10	0.25	0.00	0.46	0.79	0.80	0.92
F50	0.57	0.46	0.00	0.64	0.64	0.92
F25	0.83	0.79	0.64	0.00	0.13	0.82
F26	0.83	0.80	0.64	0.13	0.00	0.84
F32	0.93	0.92	0.92	0.82	0.84	0.00

f The Bray-Curtis dissimilarities among samples

	F9	F10	F50	F25	F26	F32
F9	0.00	1.00	1.00	1.00	1.00	1.00
F10	1.00	0.00	0.94	0.92	0.80	0.96
F50	1.00	0.94	0.00	0.97	0.67	0.95
F25	1.00	0.92	0.97	0.00	0.81	0.89
F26	1.00	0.80	0.67	0.81	0.00	0.57
F32	1.00	0.96	0.95	0.89	0.57	0.00

Fig. S3. Richness and Shannon index as well as Bray-curtis dissimilarities between samples from the HRD and DBD tanks. a-c. Bacterial communities. **d-f.** Fungal communities. Blue font indicates samples from HRD tank, red font indicates samples from diesel tank. HRD: hydro-processed renewable diesel; F76: petroleum-based Navy F76 diesel.

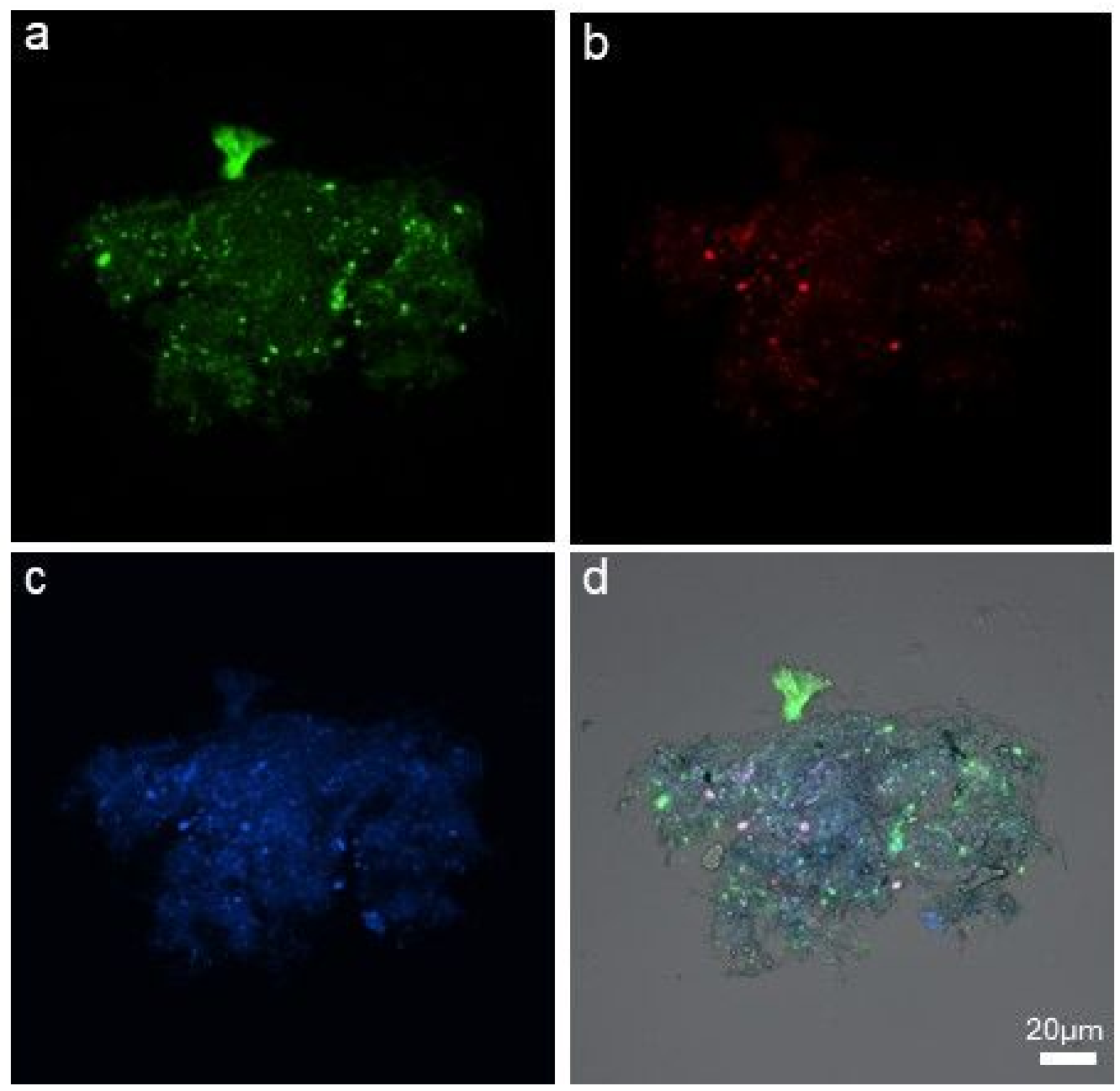

Fig. S4. Live/dead and biofilm matrix fluorescence staining of sample F50. a. Live cells stained with SYTO™9 shown in the green fluorescence channel. **b.** Dead cells stained with propidium iodide shown in the red fluorescence channel. **c.** Biofilm matrices stained with FilmTracer SYPRO Ruby, represented in blue for visual differentiation from red signal. **d.** Merged overlay of all three channels.

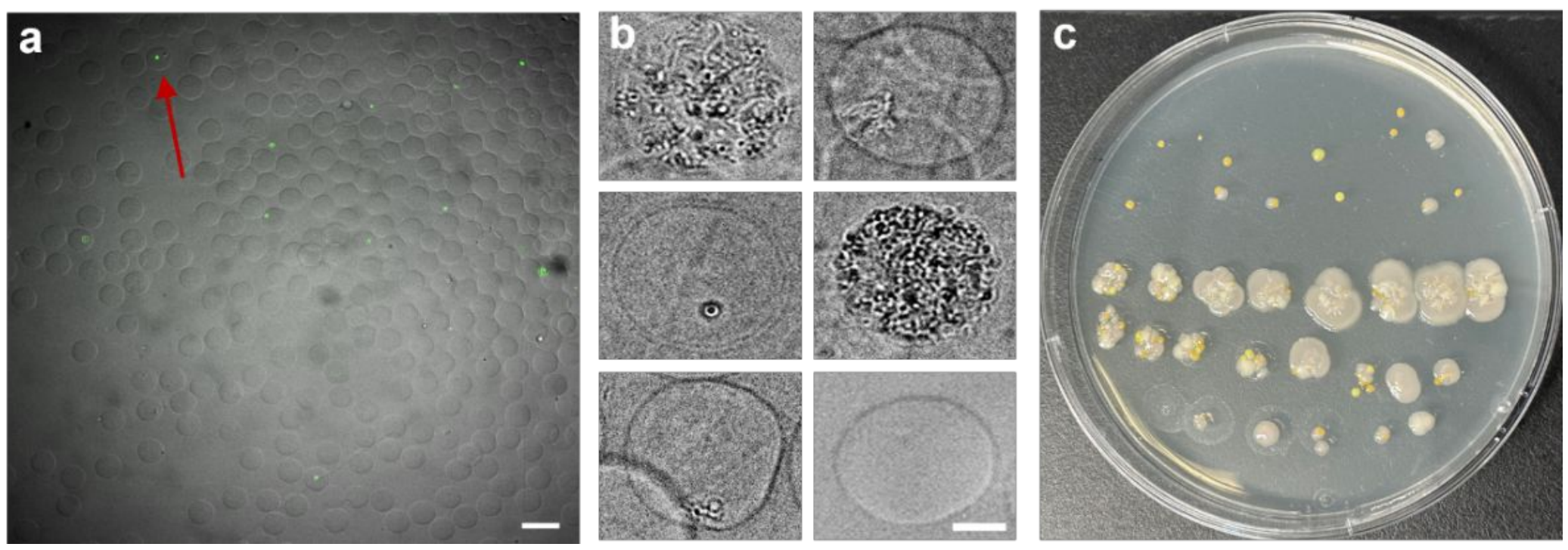

Fig. S5. Bacteria recovery using single-cell gel droplet encapsulation and culture-based technology. **a.** Single microbial cells from sample F32 (tank 2) were encapsulated into gel droplets. Red arrow indicate fluorescently stained bacteria in a gel droplet. Scale bar: 100 μm. **b.** Proliferated microbial cells in gel droplets after 48 h of incubation. Scale bar: 20 μm. **c.** Bacterial clones formed on LB plates after 72 h of incubation after cell-encapsulated gel droplets dispensed onto plates.

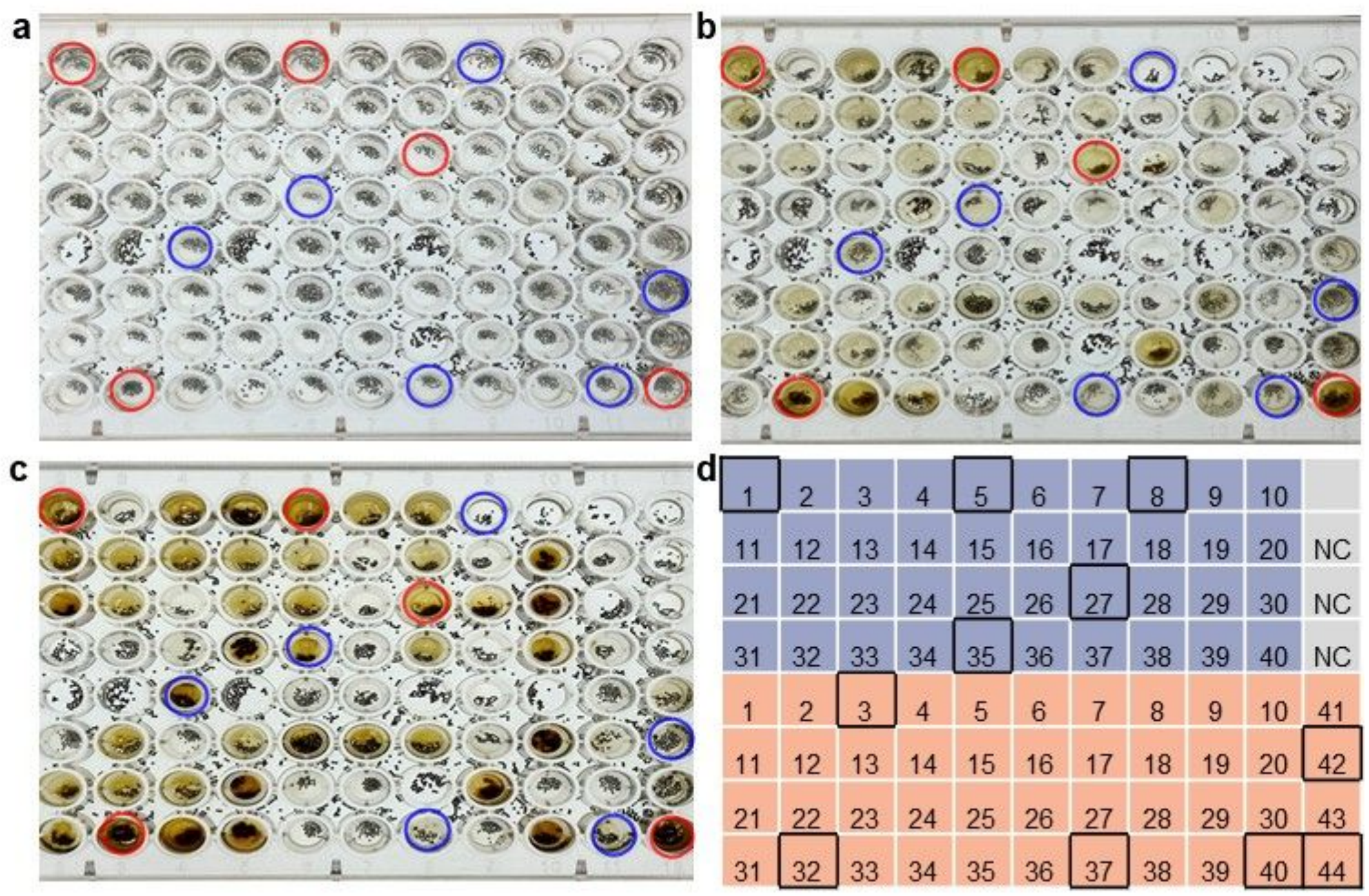

Fig. S6. Images of metal corrosion phenotype screening using CS beads in 96-well plates. In total, 88 bacterial isolates from two biofilm samples F50 and F32 were screened for their corrosivity. **a**. Time 0. **b**. After 2 days of incubation. **c**. After 7 days of incubation. **d**. Plate layout. Blue highlight: bacteria isolates from sample F50. Orange highlight: bacteria isolated from sample F32. NC: negative control R2A only without bacteria. Bordered strains represent those with a distinct corrosion phenotype and sequenced genomes. Blue circle indicates non-corrosive strain and red circle indicates corrosive strain.

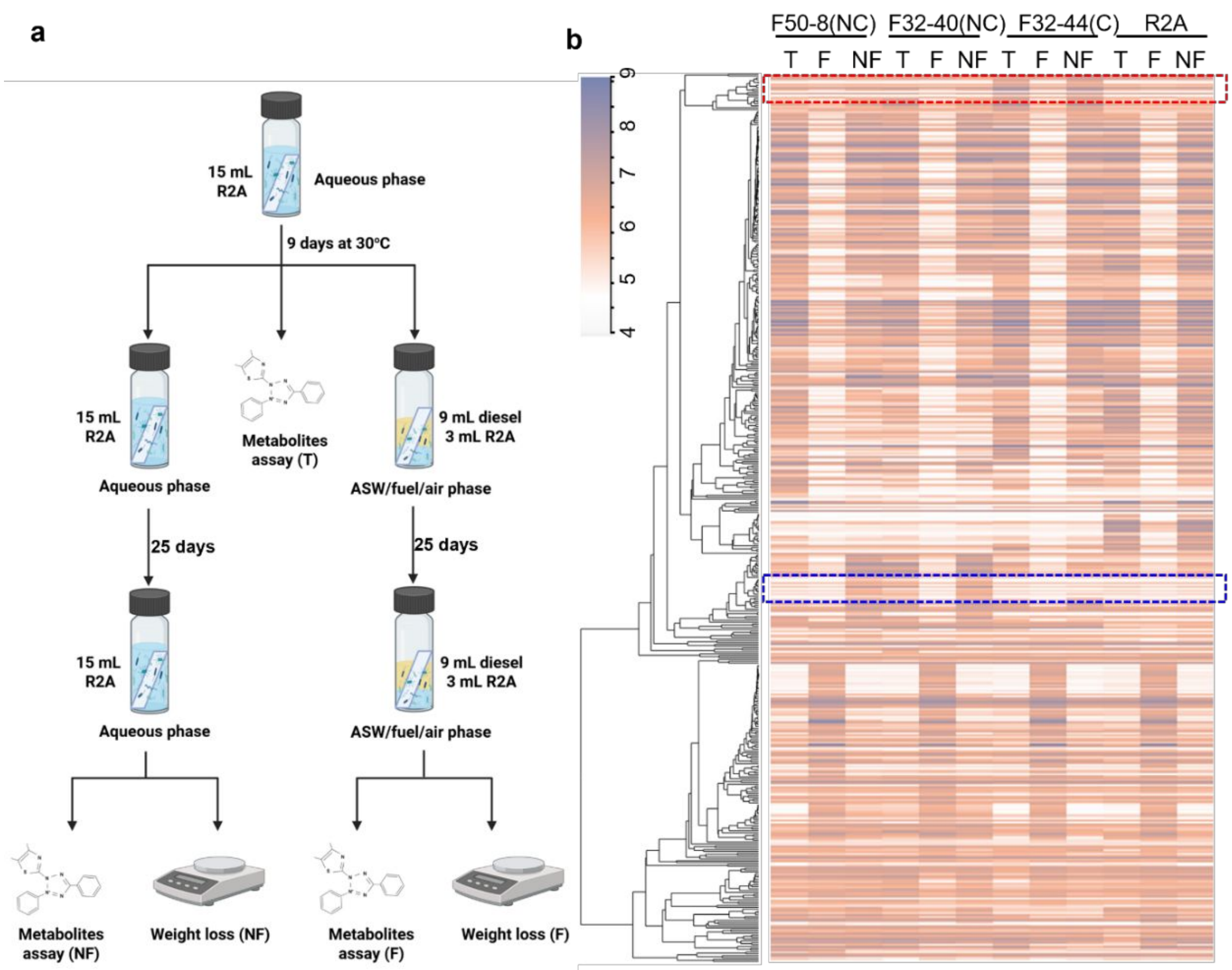

Fig. S7. Carbon steel metal coupon corrosion weight loss experiment and metabolite assay. a. Experiment flowchart. **b.** Heatmap generated by hierarchical analysis of the metabolite profiles at the beginning and end of the experiment. Suffix T indicates at the end of incubation in R2A and before being exposed to different environments; suffix F or NF indicate at the end of exposure to ASW/fuel/air conditions or R2A, respectively. N indicates negative control (R2A). Log_{10} transformed data were used for analysis. Red frame indicates metabolites only accumulated in the corrosive strain F32-44, and blue frame indicates metabolites only accumulated in non-corrosive strains F50-8 and F32-40.

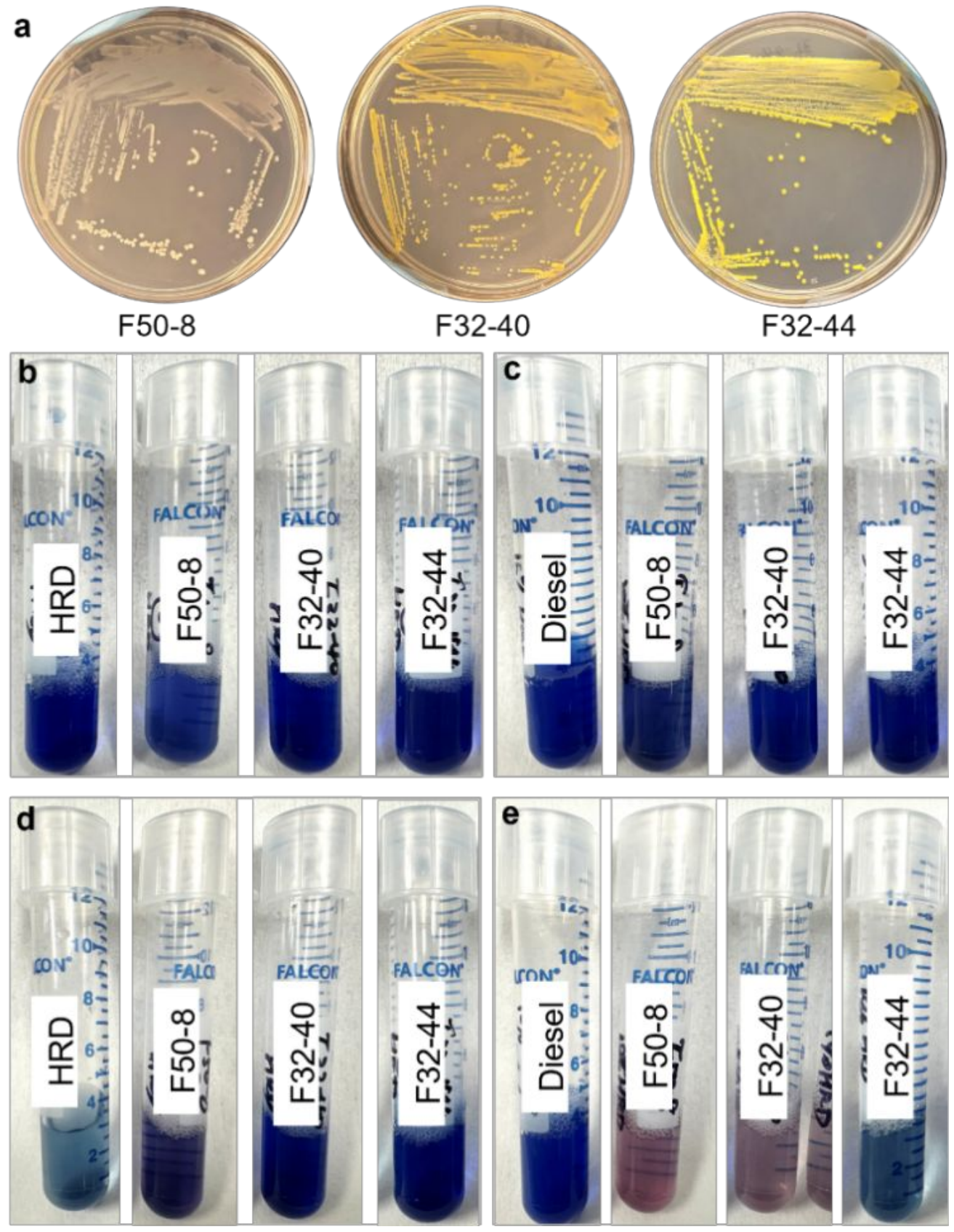

Fig. S8. Bacteria strain morphologies and growth on fuel. a. Bacteria morphologies on R2A plates. Color changes after 36 h (**b, c**) or 84 h (**d, e**). Cells were incubated in carbon-free Bushnell Haas broth medium containing 1% HRD (**b, d**) or 1% HRD-blended diesel (diesel : HRD = 9:1) (**c, e**) as carbon source with redox indicator 2,6-dichlorophenolindophenol (DCPIP, 0.05 mg/ml).

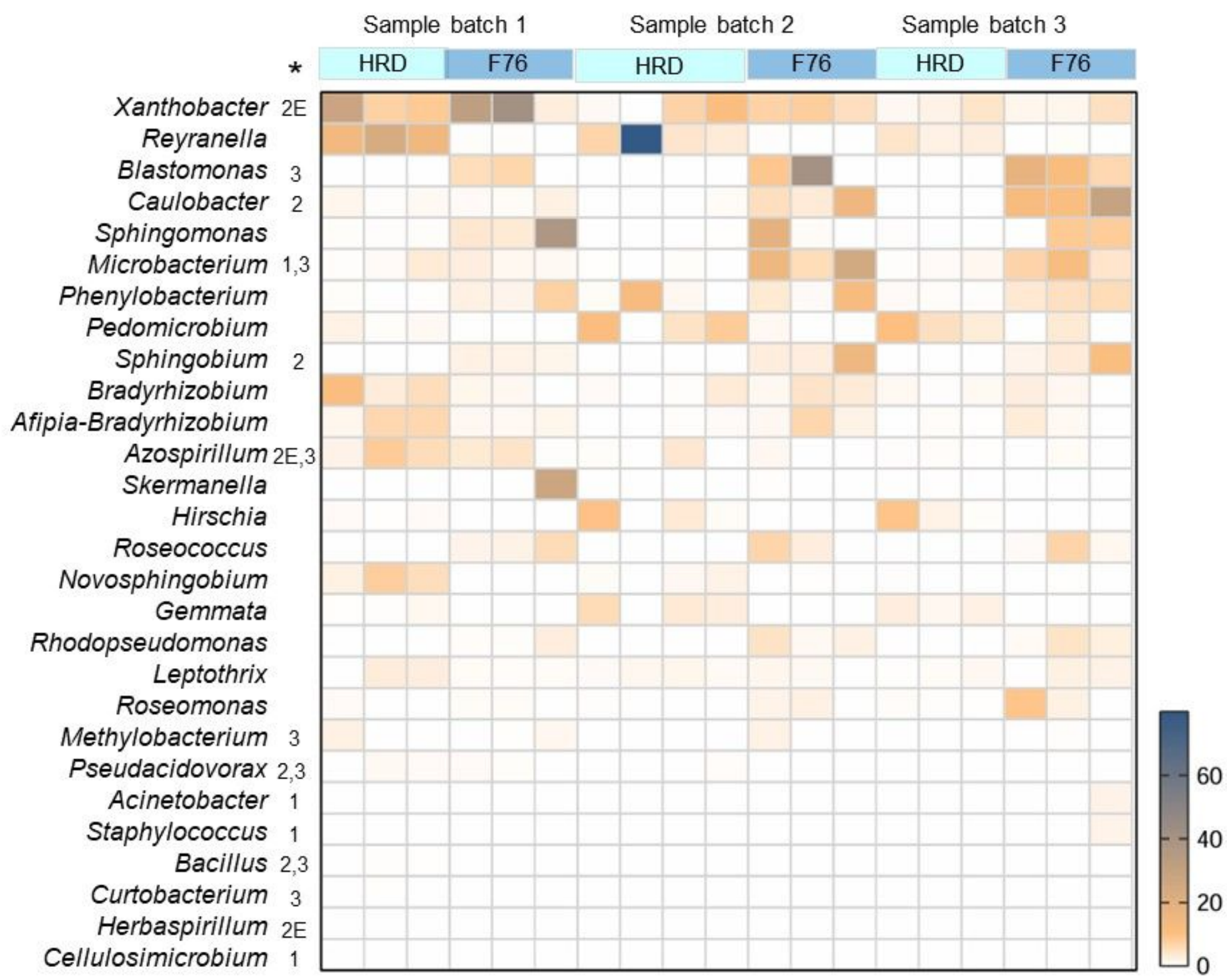

Fig. S9. Summary of the relative abundance of the top twenty most abundant genera across three sets of samples collected at different times from two tanks (HRD tank and F76 diesel tank) and the genera from which bacterial isolates were sequenced. *Column: sample batch number from which bacterial strains were sequenced. Each heatmap column represents one sample. Sample batch 1 was analyzed in this study, batches 2 and 3 were collected later from the same tanks. E indicates bacteria strains isolated from enrichment cultures.

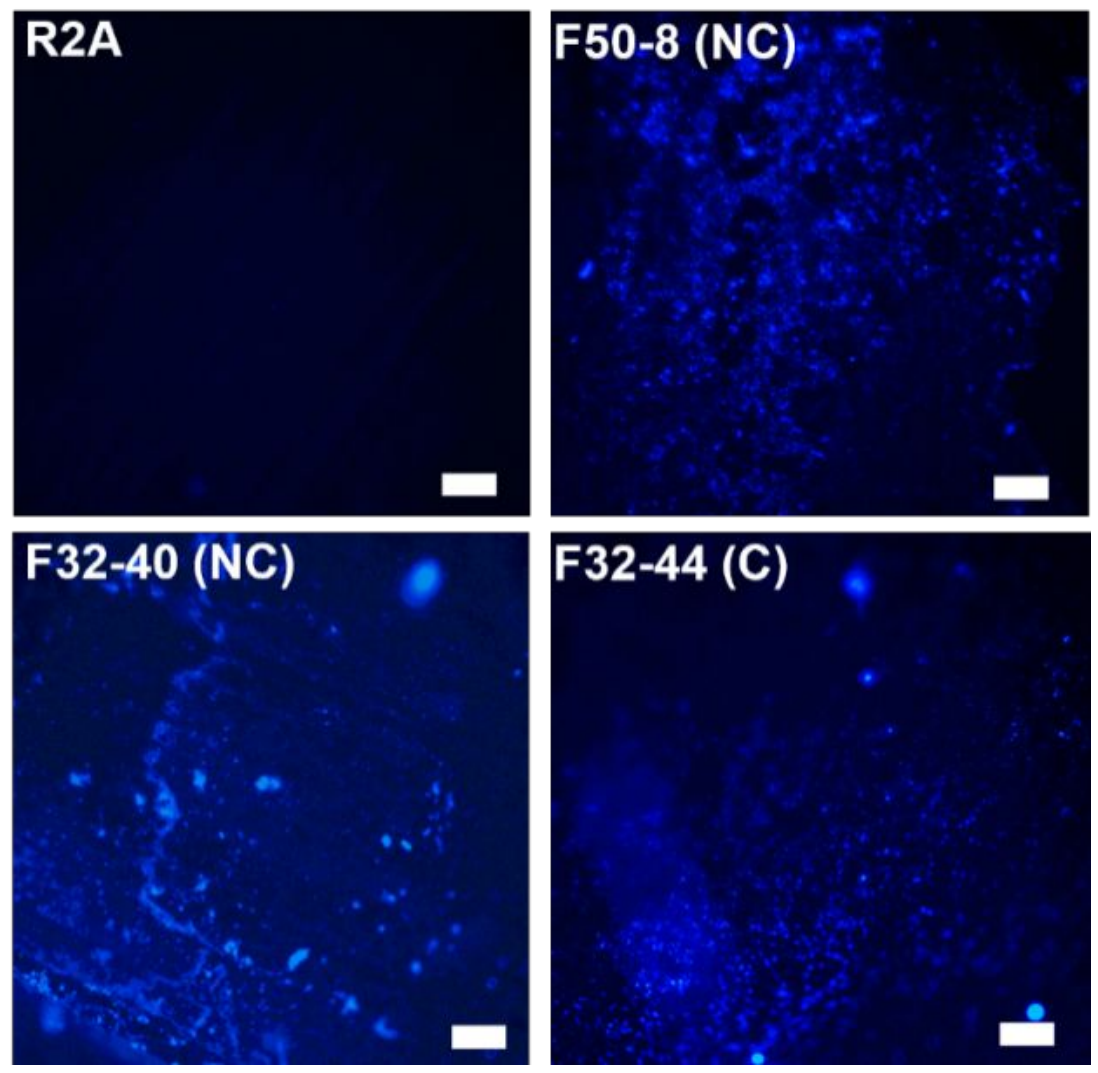

Fig. S10. Biofilms on the CS coupons stained with fluorescence dye Ruby after 9 days of incubation in R2A. Biofilm matrices stained with FilmTracer SYPRO Ruby. C: corrosive strain; NC: non-corrosive strain. Scale bar: 100 µm.

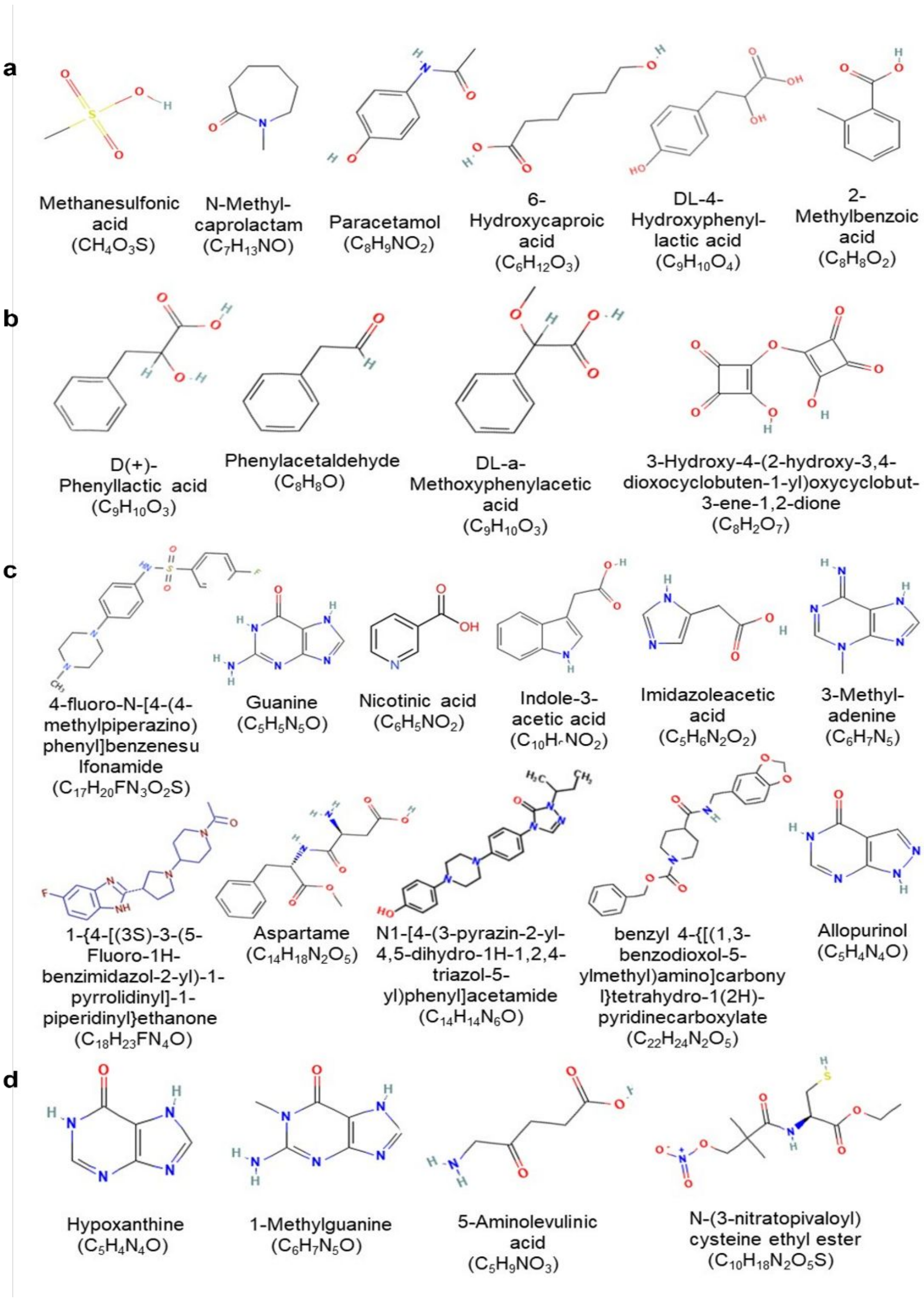

Fig. S11. Molecular structures of the key metabolites accumulated in the bacteria culture supernatants. a. Metabolites specifically accumulated in corrosive strain F32-44. **b.** Metabolites accumulated in all strains but with higher abundances in corrosive strain F32-44. **c**. Metabolites specifically accumulated in non-corrosive strains F50-8 and F32-40. **d.** Metabolites accumulated in all strains but with higher abundances in non-corrosive strains.
